# Assessing the efficacy of high-dose rifampicin plus albendazole against onchocerciasis

**DOI:** 10.1371/journal.pntd.0014594

**Published:** 2026-08-12

**Authors:** Monica Ahiadorme, Ute Klarmann-Schulz, Bettina Dubben, Jennifer Nadal, Patricia Jebett Korir, Kenneth Pfarr, Janina M. Kuehlwein, Franziska Lenz-Plet, Arcangelo Ricchiuto, Emmanuel Donawobuge Kutu, Derrick Adu Mensah, Vera Serwaa Opoku, John Boateng, John Opoku, Jubin Osei-Mensah, Charles Gyasi, Prince Obeng, Eunice Kyaakyile Kuutiero, Michael Agyemang Obeng, Benjamin Emikpe, Abu Abudu Rahamani, Prince Dennis Atisu, Nana Kwame Ayisi-Boateng, Derrick Boateng Kontoh, Sampson Twumasi-Ankrah, Achim Hoerauf, Linda Batsa Debrah, Alexander Yaw Debrah

**Affiliations:** 1 Department of Clinical Microbiology, School of Medicine and Dentistry, Kwame Nkrumah University of Science and Technology, Kumasi, Ghana; 2 Kumasi Centre for Collaborative Research in Tropical Medicine, Kwame Nkrumah University of Science and Technology, Kumasi, Ghana; 3 Department of Basic Sciences, School of Basic and Biomedical Sciences, University of Health and Allied Sciences, Ho, Ghana; 4 University of Bonn, University Hospital Bonn, Institute for Medical Microbiology, Immunology and Parasitology, Bonn, Germany; 5 German Centre for Infection Research (DZIF), partner site, Bonn-Cologne, Germany; 6 University of Bonn, University Hospital Bonn, Institute for Medical Biometry, Informatics and Epidemiology, Bonn, Germany; 7 Department of Theoretical and Applied Biology, Kwame Nkrumah University of Science and Technology, Kumasi, Ghana; 8 Department of Laboratory Technology, Faculty of Health Sciences, Kumasi Technical University, Kumasi, Ghana; 9 Department of Medical Laboratory Technology, Royal Ann College of Health, Kumasi, Ghana; 10 German-West African Centre for Global Health and Pandemic Prevention (G-WAC), partner site, Kumasi, Ghana; 11 Department of Pathobiology, School of Veterinary Medicine, Kwame Nkrumah University of Science and Technology, Kumasi, Ghana; 12 Department of Medicine, School of Medicine and Dentistry, Kwame Nkrumah University of Science and Technology, Kumasi, Ghana; 13 University Hospital, Kwame Nkrumah University of Science and Technology, Kumasi, Ghana; 14 Department of Pharmaceutics, Faculty of Pharmacy and Pharmaceutical Sciences, College of Health Sciences, Kwame Nkrumah University of Science and Technology, Kumasi, Ghana; 15 Department of Statistics and Actuarial Science, Faculty of Physical and Computational Sciences, College of Science, Kwame Nkrumah University of Science and Technology, Kumasi, Ghana; 16 German-West African Centre for Global Health and Pandemic Prevention (G-WAC), partner site, Bonn, Germany; 17 Department of Medical Diagnostics, Kwame Nkrumah University of Science and Technology, Kumasi, Ghana; University of Liverpool, UNITED KINGDOM OF GREAT BRITAIN AND NORTHERN IRELAND

## Abstract

**Background:**

Onchocerciasis, also known as river blindness, remains a public health challenge in tropical regions. Despite significant gains through mass drug administration (MDA) with ivermectin, the infection remains persistent in many endemic foci. While the disease burden has reduced, elimination may require alternative strategies, including macrofilaricidal therapies. Rifampicin (RIF) has demonstrated strong anti-*Wolbachia* activity *in vitro* and in animal models, but its clinical efficacy in humans remains uncertain.

**Methods:**

We conducted a phase II randomised, controlled trial in Ghana to evaluate the safety and efficacy of high-dose RIF (35 mg/kg/day) combined with albendazole (ALB, 400 mg/day) as an alternative treatment for onchocerciasis. The study randomised 120 infected participants into one of four arms: RIF + ALB for 14 days (TA1), RIF + ALB for 7 days (TA2), ALB alone for 14 days (TA3), and a No Treatment control group (TA4). Treatment efficacy, including *Wolbachia* and microfilariae (MF) depletion, adult worm vitality and embryogenesis were assessed following treatment at 4-, 18-, and 20-months.

**Results:**

High-dose RIF + ALB was safe and well-tolerated, associated with only mild, transient adverse events. Treatment with RIF + ALB for 14 days reduced *Wolbachia* load in skin MF at 4 months (47.9%; *p = 0.018*). ALB monotherapy showed sustained reductions in skin microfilarial densities at the 18- and 20-month time points (*p < 0.05*). However, no macrofilaricidal effect or sustained *Wolbachia* depletion in adult worms was observed in any group.

**Conclusion:**

High-dose RIF + ALB is safe with limited macrofilaricidal efficacy in clinical settings, contrasting with the superiority observed from preclinical trials. ALB alone shows promise as an alternative microfilaricidal treatment to ivermectin or as a complementary regimen. The lack of sustained efficacy of RIF as an anti-*Wolbachia* regimen may reflect suboptimal dosing, highlighting the need for further optimisation of RIF-based regimens for onchocerciasis treatment.

**Trial registration:**

PACTR202009704006025 (https://pactr.samrc.ac.za/Search.aspx). Date of trial registration: September 9, 2020

## Author summary

Onchocerciasis, commonly referred to as river blindness, is a disease caused by worms transmitted by blackflies. The disease mainly affects people living in the rural tropics, causing severe itching, skin changes, and vision loss. The main strategy for controlling the disease is through mass drug administration (MDA) with ivermectin, targeting the juvenile worms, with little or no effect on the adult worms. To complement elimination efforts, alternative drugs that can be administered within a short duration and able to kill the adult worms are urgently needed. In this study, we tested whether a combination of high-dose rifampicin (an antibiotic) and albendazole (an anti-worm medicine) could provide a better treatment option for onchocerciasis patients. We conducted a clinical trial in Ghana, enrolling 120 people with river blindness, and randomly assigning them to receive either high-dose rifampicin plus albendazole for 7 or 14 days, albendazole alone for 14 days, or no treatment. We followed participants for 20 months to assess the drugs’ safety and effectiveness in killing the juvenile and adult worms. We found that the combination of high-dose rifampicin and albendazole was safe and well tolerated, with only mild and temporary side effects. At 4 months after treatment onset, the 14-day combination treatment reduced the essential *Wolbachia* bacteria found in the juvenile worms, resident in the skin, but this effect was not observed in adult worms when assessed at 20 months. Treatment with albendazole alone also showed a reduction in the number of juvenile worms present in the skin. Regardless, none of the treatments had lasting effects that resulted in killing a significant number of the adult worms. Our results show that high-dose rifampicin plus albendazole is safe, but it does not effectively kill the adult worms or achieve long-term depletion of *Wolbachia* found in the worms. These findings highlight the need for further research to develop more effective treatments that could support the global goal of eliminating river blindness.

## Introduction

Onchocerciasis, or river blindness, is a parasitic disease caused by the filarial nematode *Onchocerca volvulus*. It remains a significant public health burden in sub-Saharan Africa, with an estimated 20.9 million people infected globally, over 99% of whom live in Africa [1,2]. The disease leads to chronic skin conditions and irreversible blindness, causing substantial social and economic hardships in affected communities [3].

Ivermectin (IVM) has been the cornerstone of onchocerciasis control and elimination through annual or biannual mass drug administration (MDA) programmes in endemic areas [4]. It effectively reduces skin microfilariae (MF) loads and transmission potential, but does not kill adult worms, necessitating repeated treatments for over a decade [4]. Although IVM-MDA has led to the elimination of onchocerciasis in some regions such as Mali and Senegal [5,6], progress remains slow in areas with intense transmission and high parasite burdens [7–9]. Moreover, its use in *Loa loa* co-endemic areas can cause severe adverse events (SAEs), including encephalopathy and death due to rapid *L. loa* MF destruction [10,11].

An extensively explored alternative is anti-*Wolbachia* therapy which targets *Wolbachia* endosymbiotic bacteria essential for the survival and reproduction of many filarial parasites, including *O. volvulus* [12,13]. Antibiotic-mediated depletion of *Wolbachia,* disrupts embryogenesis, suppresses MF production, and ultimately kills adult worms [14–16]. This macrofilaricidal activity arises from the crucial role *Wolbachia* plays in germline function; depletion of *Wolbachia* triggers widespread apoptosis in reproductive tissues, thereby blocking embryogenesis and sterilising adult worms [17]. A 4–6-week administration of doxycycline (DOX; 100–200mg/day) in onchocerciasis has shown evidence of these effects. However, this requires a prolonged treatment duration and has been shown to be contraindicated in pregnant women and children under 8 years of age, posing logistical and ethical challenges for large-scale implementation [18–21].

Recent preclinical studies have identified rifampicin (RIF), a widely used antibiotic for tuberculosis, as a potent anti-*Wolbachia* agent. A compelling rationale for combining RIF with albendazole (ALB) lies in ALB’s dual activity against both the filarial nematode and potentially, its *Wolbachia* endosymbiont. ALB is a well-established anthelmintic that disrupts nematode microtubules via *β-tubulin* inhibition. In addition, earlier cell-based and molecular studies demonstrated that its metabolite, albendazole sulfone (ALB-SO_2_), can directly reduce *Wolbachia* titres by interfering with FtsZ-mediated bacterial cell division [22,23]. Since FtsZ is encoded by *Wolbachia* and plays an essential role in cytokinesis, it represents an attractive drug target for antifilarial therapy [24]. However, in a study by Turner *et al.* (2017), administration of ALB alone had no effect on *Wolbachia* depletion, but when combined with RIF, it significantly enhances bacterial clearance [25]. Specifically, a 7-day RIF plus ALB regimen achieved >99% *Wolbachia* depletion in a *Brugia malayi* mouse model, with partial but significant reductions in female worm burden [25]. While a minimum of 14 days of high-dose RIF was required to achieve >90% *Wolbachia* depletion in male *O. ochengi* implants in an SCID murine model [12], it is worth noting that male *O. ochengi* worms are considerably smaller in biomass than adult *O. volvulus* worms, which may limit direct extrapolation of these findings to the clinical setting. These findings, nonetheless, provided the preclinical basis for the 7- and 14-day treatment durations evaluated in this current trial. Importantly, RIF at high-dose of 30–40 mg/kg is bioequivalent to the exposures used in animal models, and has been established to have favourable safety profiles in humans, including in children and pregnant women [26]. This combination therapy could potentially address the limitations of IVM and DOX, and provide a safer, more practical MDA regimen capable of achieving macrofilaricidal activity within a feasible timeframe.

Given these promising findings, it is crucial to evaluate the efficacy and safety of high-dose RIF plus ALB in human clinical trials. It is important to determine whether the combination therapy effectively kills or sterilises adult *O. volvulus* worms, suppresses or clears skin MF, and depletes *Wolbachia* in both MF and adult worms. Such evidence could provide a solid basis for the adoption of this regimen as a new tool to complement onchocerciasis elimination efforts, especially in areas where existing MDA strategies are inadequate or unsafe. This study sought to address these knowledge gaps by assessing the efficacy of high-dose RIF (35 mg/kg/day) combined with ALB (400 mg/day), administered for 7 or 14 days in a phase II randomised controlled clinical trial. The study evaluated the macrofilaricidal efficacy of different treatment regimens on adult *O. volvulus* worms, impact on embryogenesis in adult female worms, and effect on microfilaridermia. The study also assessed the effectiveness of the regimens in depleting *Wolbachia* endobacteria, using qPCR in skin MF and immunohistology techniques. In addition, the safety profile of the combination therapy was evaluated.

## Materials and methods

### Ethics statement

The study protocol, informed consent and case report forms received ethical approval from the Committee on Human Research, Publication and Ethics (CHRPE) of the School of Medical Sciences (SMS) of the Kwame Nkrumah University of Science and Technology (KNUST), Kumasi, Ghana, the Ghana Health Service (GHS) Ethics Review Committee, and the Ghana Food and Drugs Authority (FDA).

### Study population

The study was conducted in the Sefwi Akotombra district in the Western North Region of Ghana, a predominantly rural and agriculturally driven district recognised as an endemic district for onchocerciasis. The district is characterised by the Tano river and its tributaries (Yoyo, Kunuma, Suhien, and Sui), which serve as major water sources, supporting both agriculture and the local ecosystem [27]. Twenty-five (25) accessible communities were purposively selected for the trial, reflecting the distribution of the disease in the district.

Onchocerciasis control in the Sefwi Akotombra district has relied on annual mass drug administration (MDA) with ivermectin (IVM) since 1994, coordinated by the Ghana National Neglected Tropical Disease (NTD) Control Programme, and delivered through Community Drug Distributors (CDDs) or Community Health Volunteers (CHVs). In 2018, biannual MDA was introduced to intensify control efforts. However, no MDA activities were conducted in 2020 due to the COVID-19 pandemic, in accordance with WHO recommendations [28,29].

The trial was registered in the Pan African Clinical Trials Registry (PACTR202009704006025) and conducted in compliance with the Declaration of Helsinki (2013 revision) and Good Clinical Practice (GCP) guidelines. Oversight was provided by a Trial Steering Committee (TSC) and an independent Data Safety and Monitoring Board (DSMB).

Eligible participants aged 18–55 years, weighed at least 40 kg, had at least one palpable onchocercoma with confirmed positive skin MF, and were in good general health. Participants were excluded based on a history of tuberculosis (TB), intolerance to the study medications, pregnancy, breastfeeding, substance or alcohol abuse, abnormal renal and hepatic enzymes, glucosuria, hypertension, or any condition requiring long term medication.

Written informed consent was obtained from all participants, either by signature or thumbprint. Participation was voluntary, and individuals retained the right to withdraw from the study at any given time.

### Study design, sample size, randomisation and intervention

This study was a non-confirmatory randomised, open-label, phase II pilot trial, designed to evaluate the efficacy and safety of high-dose RIF combined with ALB for the treatment of onchocerciasis. As a pilot study, the sample size was determined based on precedent from similar studies, rather than on statistical argumentation. According to Julious (2005) [30], a minimum of 12 participants per treatment arm is recommended for pilot trials to provide preliminary estimates of treatment effects and variability, while considering feasibility and regulatory guidance.

The study enrolled and randomised 120 participants into four treatment arms (30 per arm), accounting for a 30% dropout rate, consistent with previous onchocerciasis clinical trials. Treatments were administered under directly observed therapy (DOT) by the trial clinician and pharmacist within participants’ communities, beginning March 8, 2021. RIF and ALB were administered simultaneously, and participants were encouraged to avoid alcohol intake within the treatment period and to eat prior to taking the study medications to improve gastrointestinal tolerance, support treatment adherence, and ensure optimum absorption [31–33]. The treatment duration corresponded to the assigned arm, with a maximum of 21 days allowed to accommodate any missed doses.

The treatment arms were as follows:

- **Treatment Arm 1 (TA1)**: Rifampicin 35 mg/kg/day plus albendazole 400 mg/day for 14 days
- **Treatment Arm 2 (TA2)**: Rifampicin 35 mg/kg/day plus albendazole 400 mg/day for 7 days
- **Treatment Arm 3 (TA3)**: Albendazole 400 mg/day for 14 days
- **Treatment Arm 4 (TA4)**: No treatment (control)

The RIF-based regimens were informed by prior evidence supporting short-course, high-dose therapy [12,25] with ALB added to enhance antifilarial activity and to assess the feasibility of a shorter-course treatment approach [25,34]. RIF monotherapy was not included, as evidence indicates superior efficacy with combination regimens [34–36], and the additional arms were not feasible due to logistical and ethical constraints. An ALB monotherapy arm was included to reaffirm its potential role in onchocerciasis treatment, supported by findings that standard-dose ALB produces sustained reductions in microfilarial densities [37,38].

Participants and the study team were not blinded to the treatment allocations. However, outcome assessors conducting histological and qPCR analyses were blinded to minimise assessment bias.

All participants were actively monitored and assessed by the trial clinician during and after administration of study medications for the occurrence of adverse events (AEs). All study procedures were conducted in accordance with GCP guidelines to ensure the safety and rights of participants were safeguarded throughout the study period. Participants were followed up at 4-, 18- and 20-months post-treatment to assess treatment efficacy. IVM was administered, 6 months following treatment with RIF and ALB to all groups, including the no treatment arm. This was done in accordance with ethical considerations and standard of care.

### Study outcome measures

The primary outcome of the trial was the proportion of viable adult *O. volvulus* worms in accessible nodules assessed by histology at 20 months post-treatment. Secondary outcomes included the assessment of embryogenesis within female adult worms, presence of free MF within nodules by histology at 20 months post-treatment, absence of *Wolbachia* endobacteria in skin MFs assessed by qPCR at 4-months post-treatment, qualitative and quantitative evaluation of *Wolbachia* depletion in adult worms at 20 months post-treatment using immunohistology and qPCR, respectively, and the presence or absence of skin MFs, detected by skin snip microscopy.

### Parasitological assessment

All enrolled participants presented with at least one palpable nodule (onchocercoma). Skin MF loads were assessed using the standardised skin snip protocol, in which two bloodless 2-mm skin biopsies were taken from the left and right iliac crests and incubated overnight at room temperature in 1 mL of sterile physiological saline to allow MF emergence [39]. MF density was calculated as the arithmetic mean number of MF per mg skin snip. Two independent microscopists, blinded to treatment allocations, performed MF enumeration, and the mean of their counts was used for analysis [39].

### Nodulectomy and histological assessment

At 20 months post-treatment, palpable nodules were surgically removed from consenting participants at the Sefwi Wiawso Government Hospital under aseptic conditions and local anaesthesia. Each nodule was labelled with participant identifiers, fixed in 80% ethanol, and processed using an STP-120 automated tissue processor (Microm International GmbH, Thermo Fisher Scientific, Walldorf, Germany) before embedding in paraffin wax. Serial sections of 5 µm were cut from the paraffin blocks using a pfm Rotary 3005E microtome (pfm medical gmbh, Cologne, Germany) for histological, immunohistological, and molecular analyses following established protocols.

A minimum of 8 sections per nodules were assessed by two experienced parasitologists blinded to the treatment allocations. For histology, sections were stained with Haematoxylin and Eosin (H&E; Merck, Darmstadt, Germany) to evaluate worm morphology including sex differentiation, embryogenesis, and the presence of MFs within nodular tissue. Iron deposits in worm tissues were detected using the Pearls Prussian Blue method with potassium ferrocyanide-hydrochloric acid solution and nuclear fast red counterstain [40], where the intensity of blue staining of the worm gut correlated with worm age: strong staining indicated older worms, light staining for younger worms, and absence of staining suggested dead worms. Worms lacking conclusive iron staining or those decalcified with EDTA were classified as not judgeable.

For immunohistological detection of worm vitality and *Wolbachia* endobacteria, the Dako REAL Detection System (Alkaline Phosphatase/RED, Labelled Streptavidin-Biotin (LSAB) method; Dako Cytomation, Hamburg, Germany) was employed. A rabbit anti-serum against *Dirofilaria immitis Wolbachia* surface protein (DiWsp) at a dilution of 1:2000 with REAL antibody diluent was used to detect *Wolbachia* [41]. Secondary detection used a REAL Biotinylated Secondary Antibody (AB2), followed by REAL Streptavidin Alkaline Phosphate (AP) labelling, with REAL Chromogen Red as the chromogen, and REAL Haematoxylin as counterstain. Additionally, a rabbit anti-serum against a cathepsin D-like lysosomal aspartic protease of *O. volvulus* (APR) at 1:2000 dilution with REAL antibody diluent was employed to assess worm vitality and estimate age: absence of APR staining, calcification, loss of cuticle, or organ degeneration indicated dead worms [42], while strong APR positivity in hypodermis indicated living worms [43,44], with staining intensity further used to classify age as newly acquired, or old [45]. Moribund worms with extensive degeneration but APR-positivity were classified as dead, especially if associated with neoplasms. Stained slides were digitalised at 20X magnification using a Zeiss AxioScan (Carl Zeiss, Jena, Germany) and analysed with ZEN 3.10 software (blue edition).

*Wolbachia* content was classified as ‘none’ (no visible staining), ‘few’ (less than 50 bacteria per hypodermis) and ‘many’ (densely packed bacteria), applicable to both adult worms and embryos [45]. Embryogenesis was evaluated by identifying developmental stages from oocytes to stretched MF in the uterus; normal embryogenesis required the presence of all stages [46,47].

### DNA extraction from microfilariae and nodule sections

Genomic DNA was extracted from skin MF and paraffin-embedded nodule sections using the QIAamp DNA Micro Kit (50) [48,49], following the protocol described by Schlabe *et al.* (2022) [50]. For nodule samples, 8 sections (5 µm thickness) per nodule were processed. The same extraction procedure was used, except that nodule sections underwent an overnight proteinase K digestion at 56 ^°^C to ensure complete tissue lysis. DNA was eluted in AE buffer and stored at -20 ^°^C until further analysis.

### Duplex qPCR assay

Quantification of *Wolbachia*-specific DNA in samples extracted from skin MFs and nodule sections was performed using a duplex qPCR assay targeting the *Wolbachia* single-copy gene *ftsZ* (*wOvftsZ;* GenBank: AJ276501) and the *O. volvulus* housekeeping gene actin (*OvActin*; GenBank: M84916.1), following the protocol described by Schlabe *et al.* (2022) [50]. The primer and probe sequences used in the assay are provided in S1 Table. The assay was run on a RotorGene 6000 (Qiagen, Hilden, Germany). *Wolbachia* copy number was normalised per MF (*wOvFtsZ* copy number/μL x elution volume ÷ number of MF) or relative to *OvActin* in nodule sections (wOvFtsZ copy number/μL ÷ OvActin copy number/μL) to account for variability in worm material across samples. Positive controls from our previous clinical trial (MoRion, ISRCTN43697583) were included in all assays runs to monitor qPCR performance and reproducibility.

### Statistics

All statistical analyses were performed using Stata version 16 (Stata Corporation LLC, College Station, USA) and SAS version 9.4 (SAS Institute Inc., Cary, NC, USA). Data visualisation was performed using GraphPad Prism 10.2 (GraphPad Software, Inc., San Diego, CA).

Continuous variables were assessed for normality using the Shapiro-Wilk test. Unless where stated, non-parametric tests were used for analysis. Analysis of baseline characteristics of participants included the Fisher’s exact test for sex and IVM rounds (grouped); Kruskal-Wallis test for number of IVM rounds, nodule counts, nodule sites and MFs/mg skin; and analysis of variance (ANOVA) for age, body weight and years lived in an endemic area.

In analysing treatment efficacy, three datasets were utilised:

1. **Per protocol (PP):** Participants who completed treatment per the protocol.
2. **Intention-to-treat (ITT):** Participants who took the drugs at least once.
3. **MF-PP:** Skin snipped participants who were treated PP, and present at 4-, 18- and 20-months post-treatment and had taken IVM at the 6-months follow-up.

Given that the study was a non-confirmatory pilot trial, efficacy analysis primarily relied on the PP dataset. The ITT dataset was used to describe baseline characteristics, assess adverse events, and corroborate findings from the PP analyses. For nodulectomy-based histological outcomes, all individuals who underwent the procedure were included in the ITT analysis, without imputation for missing values.

Histological evaluation endpoints were analysed using alternating logistic regression, implemented in SAS-Procedure Genmod to account for potential dependencies among worms within a patient. The Odds Ratios (ORs) with 95% confidence intervals (CIs) were derived from regression models to assess differences between treatment groups regarding outcome measurements (occurrence of dead worms, *Wolbachia* depletion, wOvFtsZ, OvActin, wOvFtsZ/OvActin ratio, inhibition of embryogenesis).

In comparing treatment groups for MF-PCR, data analysis was performed using the Kruskal-Wallis test, followed by Dunn’s post hoc test for multiple comparisons, with the Holm-Bonferroni correction applied.

Within treatment (before and after treatment) differences in microfilarial loads (MF/mg skin snip) were assessed using the Wilcoxon-signed-rank-test. A p-value < 0.05 was considered significant.

## Results

### Participants flow and recruitment

**Fig 1:**
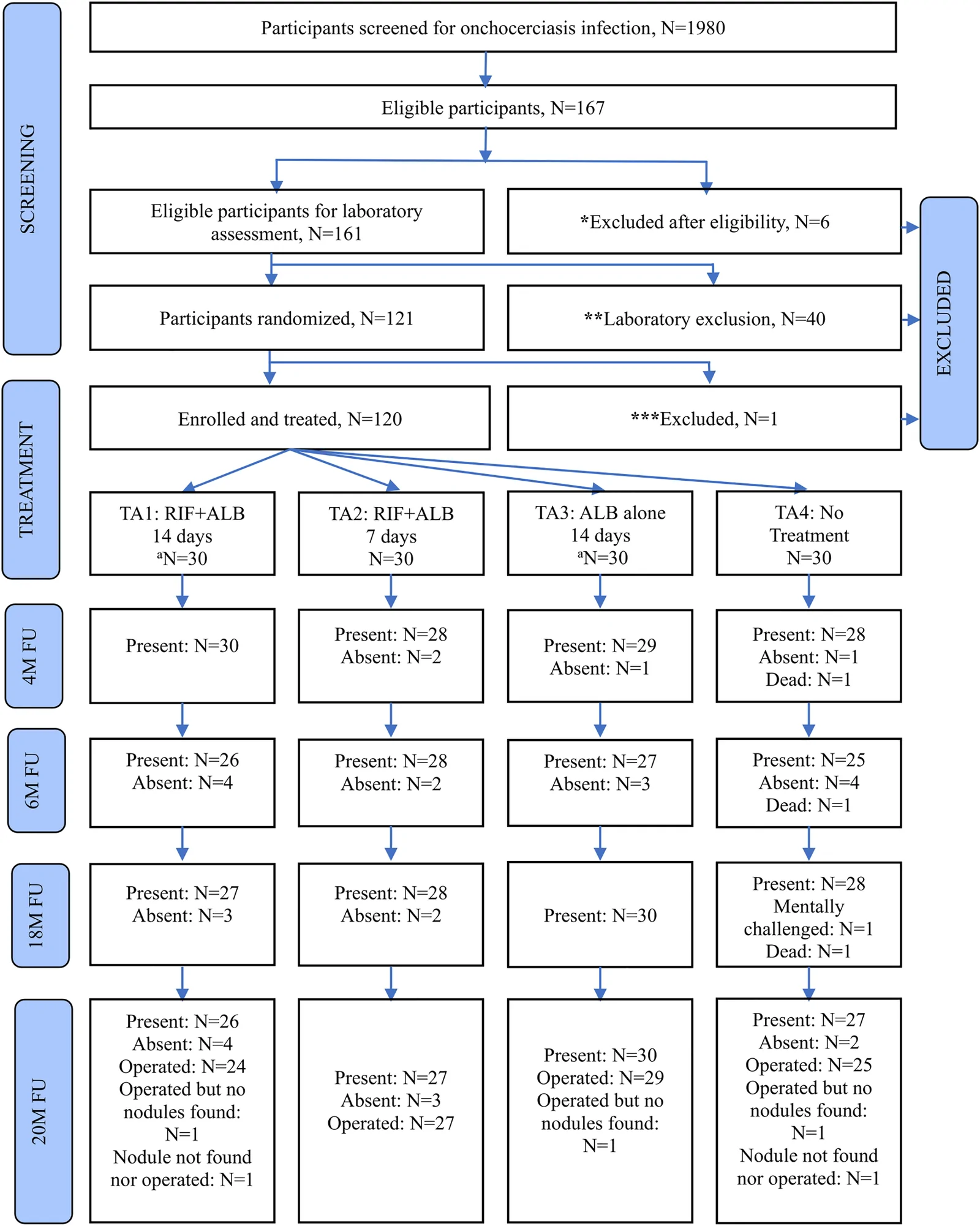
CONSORT diagram showing the number of participants screened, randomised, treated, followed up and nodulectomised. *Mentally ill (N = 1), Refused (consent withdrawn) (N = 3), Absence of medium-sized nodule (N = 1), Age > 55yrs (N = 1). **Pregnancy (N = 1), High GGT only (N = 8), High GGT and Epileptic (N = 1), eGFR < 60 only (N = 3), eGFR < 60 and Pregnancy (N = 1), Low WBC (N = 1), High AST and GGT (N = 2), High ALT and GGT (N = 1), High AST and ALT (N = 1), High AST, ALT and GGT (N = 2), Absence of medium-sized nodule (N = 14), Excluded due to refusal to continue in the study before randomisation and treatment (N = 1), eGFR < 60 and small nodule (N = 1), Pregnant and small nodule (N = 1), High GGT and small nodule (N = 2). ***Met inclusion criteria and randomised but later excluded due to refusal of treatment (N = 1). The participant never received the first dose of treatment.. ^*a*^*Failed to complete treatment due to: High AST and ALT; TA3 (N = 1), High AST and low eGFR; TA1 (N = 1).*Reasons for missing participants at follow-ups: *4-months: TA2- travelled (N = 2), TA3- travelled (N = 1), TA4- travelled (N = 1) and dead (N = 1). 6-months: TA1- travelled (N = 4), TA2- travelled (N = 2), TA3- travelled (N = 3), TA4- travelled (N = 4) and dead (N = 1). 18-months: TA1- travelled (N = 3), TA2- travelled (N = 2), TA4- mentally challenged (N = 1) and dead (N = 1). 20-months: TA1- travelled (N = 4), TA2- travelled (N = 3), TA4- travelled (N = 2) and dead (N = 1). N = number; TA = Treatment arm; GGT = gamma-glutamyl transferase; AST = aspartate transaminase; ALT = alanine transaminase; eGFR = estimated glomerular filtration rate*.

A total of 120 participants were enrolled from 25 villages in the Sefwi Akontombra district and randomised 30 per treatment arm into 4 groups as detailed in the study flow chart (Fig 1). All participants received one of the four assigned treatments, and underwent follow-up evaluations at 4-, 18- and 20 months post-treatment. Participant recruitment and enrolment occurred between February 10 and March 8, 2021. Treatment was administered from March 8 to April 4, 2021, and follow-up was completed on December 7, 2022.

### Baseline characteristics

Participants enrolled and treated had at least one palpable nodule and a baseline skin snip positivity for MF. Baseline characteristics showed no statistical differences between treatment arms for most variables, including sex, body weight, number of IVM rounds, IVM rounds (grouped), nodule counts, nodule site (location), and microfilaridermia intensity (MF/mg skin snip) (Table 1). However, age (p = 0.030) and years lived in the endemic area (p = 0.010) differed between the 4 arms, which occurred randomly during the treatment assignment process. Approximately 12% of participants reported no prior IVM consumption at study onset, but this did not differ between treatment groups or had any effect on treatment outcomes (Table 1).

**Table 1:** Baseline characteristics of participants.

		RIF + ALB – 14 Days (TA1)	RIF + ALB – 7 Days (TA2)	ALB alone – 14 Days (TA3)	No Treatment – Control (TA4)	P-value
**Baseline**						
Participants	N	30	30	30	30	
Sex	Male, N (%)	21 (70.0)	21 (70.0)	21 (70.0)	21 (70.0)	1.000^a^
	Female, N (%)	9 (30.0)	9 (30.0)	9 (30.0)	9 (30.0)	
Age (years)	Mean (±SD)	41.00 (9.14)	35.13 (10.08)	40.83 (10.60)	35.43 (10.54)	0.030^b^
	Min-Max	26-55	18-50	19-55	18-52	
Years in endemic area	Mean (±SD)	26.20 (9.56)	23.17 (9.56)	29.13 (11.26)	20.87 (8.93)	0.010^b^
	Min-Max	4-50	3-50	10-54	4-40	
Body Weight (kg)	Mean (±SD)	57.44 (8.22)	57.27 (9.39)	57.54 (7.81)	57.95 (8.33)	0.991^b^
	Min-Max	40.0-85.0	45.0-76.0	45.0-75.1	45.0-77.0	
Number of IVM rounds	Median	3	4	3	4	0.820^c^
	Min-Max	0-6	0-7	0-8	0-6	
	Percentiles 25^th^- 75^th^	3.00;4.00	3.00; 4.00	3.00;4.00	2.00; 4.00	
IVM rounds (categorised)	0 round N (%)	2 (6.67)	3 (10.00)	5 (16.67)	4 (13.33)	0.745^a^
	1-4 rounds N (%)	24 (80.00)	21 (70.00)	18 (60.00)	22 (73.33)	
	≥ 5 rounds N (%)	4 (13.33)	6 (20.00)	7 (23.33)	4 (13.33)	
Nodule counts	Median	2	2	2	2	0.552^c^
	Min-Max	1-6	1-7	1-8	1-4	
	Percentiles 25^th^- 75^th^	1.00; 3.00	1.00; 3.00	1.00; 3.00	1.00; 3.00	
Nodule Sites	Median	2	2	1	1	0.794^c^
	Min-Max	1-3	1-3	1-3	1-3	
	Percentiles 25^th^- 75^th^	1.00;2.00	1.00;2.00	1.00;2.00	1.00;2.00	
MF/mg skin snip	Median	0.70	0.83	1.06	0.77	0.581^c^
	Min-Max	0.06-15.75	0.08-15.81	0.10-64.73	0.06-34.71	
	Percentiles 25^th^- 75^th^	0.33;1.17	0.33;2.87	0.36;3.58	0.25;1.94	

*TA = Treatment arm; RIF = Rifampicin; ALB = Albendazole; TA = Treatment arm; N = Number; SD = Standard deviation; CI = Confidence interval*

*^a^Fisher’s exact test;* ^*b*^*Analysis of variance (ANOVA) test;* ^*c*^*Kruskal-Wallis test*

*Statistics calculated using Stata version 16*

### Adherence to treatment and post-treatment follow-ups

High treatment adherence was observed, with 98.3% (118/120) of enrolled participants completing their assigned treatment regimens per protocol. All participants, including those in the control group/no treatment arm (TA4), were monitored for adverse reactions during the 15-day treatment phase, with a 6-day grace period for missed doses.

Post-treatment follow-ups occurred at 4-, 6-, 18-, 20-month intervals to assess therapeutic efficacy. Out of the 120 participants, retention rates at the four time points were 95.8% (115) at 4 months, 89.1% (106) at 6 months, 95% (113) at 18 months, and 92.4% (110) at 20 months. Skin snip samples were collected at all time points except for the 6-month visit, which focused on IVM administration.

At the 6-month follow-up, IVM was administered to 97.2% (103/106) of the eligible participants. At the 20-month follow-up, 98.2% (108/110) of participants underwent nodulectomy, with two excluded due to absence of palpable nodules. Nodules were successfully excised from 97.2% (105) of participants who consented to the procedure, while no palpable nodules were found during surgery in the remaining 3 participants.

### Adverse events following treatment

Adverse events (AEs) were carefully monitored throughout the treatment period to ensure participant safety. RIF and ALB were generally well tolerated, with most AEs classified as mild to moderate. Despite the occurrence of adverse reactions, nearly all participants completed the assigned regimens, with treatment discontinued in two participants due to safety concerns. In addition, seven participants missed one or two doses; however, all completed treatment within the 21-day period. Missed doses were attributable to participant unavailability rather than AE-related complications. Detailed information on treatment interruptions and discontinuation is provided in S2 Table. During treatment, 38 (31.7%) out of the 120 participants recorded AEs (S3 Table). The highest number of participants experiencing AEs was observed in the RIF + ALB – 7 Days group (TA2), where 18 (60%) reported at least one AE. Notably, only one participant (3.3%) in the No Treatment/Control arm (TA4) reported an AE, specifically, elevated AST, despite not receiving any study medication (S3 Fig). A total of 50 AEs were reported, with the highest occurrence in the RIF + ALB – 7 Days group (TA2) at 44.0% (22 AEs), followed by RIF + ALB – 14 Days group (TA1) at 42.0% (21 AEs) (S4 Table).

Overall, 14 different types of AEs were identified. The most frequent AE was reddish-coloured urine (58.0%, 29/50), attributed to RF’s characteristic discoloration. Other common AEs included headache (8.0%, 4/50), fever (4%, 2/50), and elevated levels of ALT (6.0%, 3/50), AST (4%, 2/50) and GGT (4%, 2/50). Less frequent AEs included nausea, diarrhoea, stomach pain, and runny nose. In addition, one participant developed ulceration of the hand and face due to a motor accident, which was classified as a non-treatment related adverse event. Other laboratory abnormalities included low HB (<8 g/dL), low WBC (<3 × 10^6^/µL), and low eGFR (<60 mL/min/1.73m²). Participants were closely monitored until all AEs were resolved.

One serious Adverse Event (SAE) was recorded during the study: a participant in the no treatment arm (TA4) was reported to have committed suicide during the 4-months follow-up. This event was deemed unrelated to the study medications.

## Immunohistology

### Live versus dead worms

A total of 191 nodules from 89 participants **(PP,** S5 Table**),** and 223 nodules from 105 participants **(ITT,** S6 Table) were examined. In all, 12.0% (23/191) of nodules could not be assessed for viability because they were of non-onchocercal origin (foreign body granulomas, lipomas, lymph nodes). Hence, 168 nodules from 79 participants **(PP,** S5 Table), and 196 nodules from 92 participants **(ITT,** S6 Table) were used in subsequent analyses.

The proportion of dead female worms varied across treatment groups, ranging from 25.0% (23/92) in the RIF + ALB – 14 days (TA1) to 36.2% (38/105) in the No Treatment/Control (TA4) group. Statistical analysis using alternating logistic regression (SAS Proc Genmod), which accounts for clustering of worms within patients, revealed no significant differences in the proportion of dead versus live female worms between treatment groups (PP: p = 0.184; ITT: p = 0.219). This analysis accounted for potential dependencies between worms within the same patient, which can arise due to shared host environment and treatment exposure (S5 and S6 Tables**).**

On average, each nodule contained a total of 2.42 female worms—2.40 living and 2.48 dead female worms for the PP analysis, while the ITT analysis showed an average of 2.30 female worms—2.28 living and 2.38 dead female worms.

For male worms, a lower proportion of mortality was recorded across treatment arms, ranging from 2.8% to 9.7%, lower than the rate reported for female worms. The highest proportion of dead male worms (9.7%, 3/31) was found in the RIF + ALB – 14 days (TA1) group, and the lowest (2.8%, 1/36) in the RIF + ALB – 7 days (TA2) group. As observed for female worms, and accounting for potential dependencies between worms within the same patient, statistical analysis using alternating logistic regression in SAS Proc Genmod revealed no significant differences in the proportion of dead male worms across groups (PP: p = 0.872; ITT: p = 0.838) (S5 and S6 Tables).

On average, each nodule contained a total of 1.15 male worms—1.16 living and 1.00 dead male worms in the PP analysis, while the ITT analysis reported an average of 1.14 male worms—1.15 living and 1.00 dead male worms.

### Inhibition of embryogenesis

The study evaluated the effect of the treatments on *O. volvulus* embryogenesis by comparing the proportions of adult female worms with normal and degenerated embryos across treatment groups. The impact of the treatment regimens on embryogenesis was evaluated using an alternating logistic regression model (S8 and S10 Tables). The proportions of living female worms with normal embryogenesis were 28.6% (18/64), 32.9% (24/79), 32.7% (16/58) and 30.5% (18/63) in the RIF + ALB – 14 Days (TA1), RIF + ALB – 7 Days (TA2), ALB alone – 14 Days (TA3) and No Treatment/Control (TA4) groups (S7 Table).

Alternating logistic regression analysis showed no statistical difference in embryogenesis between the treated groups (TA1, TA2, TA3) and the control group (TA4) (p > 0.05) (S8 Table**).** However, the RIF + ALB – 7 Days (TA2) and the ALB alone – 14 Days (TA3) groups had a 7.9% and 7.2% reduction in the proportion of female worms with normal embryogenesis, compared to the No Treatment/Control group, respectively. Similarly, the RIF + ALB – 14 Days (TA1) group showed a 6.2% reduction in the proportion of female worms with normal embryogenesis compared to the No Treatment/Control group.

In addition to embryogenesis, another indicator of female worm fecundity—the presence of free MF in nodule tissues—was also evaluated. Like the findings on embryogenesis, no differences were observed between the four treatment groups (p = 0.571, S11 Table).

Both PP and ITT analyses showed no statistically significant differences between the treatment groups and the control group, confirming the robustness of the primary analysis and minimising potential bias due to non-compliance with the treatment regimen or loss to follow-up after treatment initiation (S9–S10 Tables**).**

### Effect of study medications on *Wolbachia* depletion

Th*e Wolbachia* loads in living *O. volvulus* were assessed semi-quantitatively and classified as ‘many’, few’ and ‘none’ as previously described [47]. In total, 168 nodules from 79 individuals were analysed in the PP group (S14 Table**).** In the ITT group, 196 nodules from 92 study participants were analysed (S15 Table**).** The proportion of living female worms containing *Wolbachia* was not reduced in any of the treatment arms compared to the No Treatment/Control arm (S14 and S15 Tables**).** Even though 4.8% of living female worms in the RIF + ALB – 14 Days (TA1) had no *Wolbachia* (S14 Table), statistical analysis revealed no significant reduction in *Wolbachia* load in this group compared to the control group, where 1.6% of female worms had no *Wolbachia*.

For male worms, there was no statistical difference in *Wolbachia* reduction in the treated arms when compared to the No Treatment/Control arm for male worms that could be assessed for *Wolbachia* (S16 and S17 Tables**).** The *Wolbachia* assessment methods were the same for both male and female worms.

## Real-time quantitative PCR (qPCR)

### *Wolbachia* depletion in skin microfilariae

Traditionally, anti-*Wolbachia* drug efficacy evaluations have relied on immunohistological examination of nodulectomised samples, a method limited by its invasiveness and single time point assessment throughout a study. Real-time quantitative PCR (qPCR) targeting *Wolbachia* DNA in skin MF offers a less invasive surrogate, enabling longitudinal monitoring of *Wolbachia* depletion and early identification of sub-optimal drug responses in a study.

In this study, qPCR assays amplified the single-copy *Wolbachia* ftsZ gene (wOvFtsZ) and the *O. volvulus* actin gene (OvActin). The ratios wOvFtsZ/MF and wOvFtsZ/OvActin were calculated to assess treatment efficacy at 4-months post-treatment. Samples were included if microscopy confirmed at least 1 MF count and OvActin was detected by qPCR. Four MF-positive samples (microscopy) that were OvActin negative across the treatment arms except the no treatment/control arm (TA4) were excluded from the *Wolbachia* depletion analysis (Table 2).

**Table 2:** Selection of 4-months skin snip samples for qPCR and *Wolbachia* depletion analysis—PP analysis.

		RIF + ALB – 14 Days (TA1)	RIF + ALB – 7 Days (TA1)	ALB alone – 14 Days (TA3)	No Treatment/ Control (TA4)	P-value
**Baseline**						
Participants	N	29	30	29	30	
Skin snips	Total, N	58	60	58	60	
MF Positive	N (%)	44 (75.9)	52 (86.7)	53 (91.4)	51 (85.0)	0.141^a^
**4 Months**						
Participants	N	29	28	28	28	
Skin snips	Total, N	58	56	56	56	
MF Positive	N (%)	40 (69.0)	42 (75.0)	43 (76.8)	49 (87.5)	0.112^a^
OvActin Positive^†^	SS^†^, N (%)	39 (97.5)	40 (95.2)	42 (97.7)	49 (100.0)	0.416^a^
*w*OvFtsZ Positive^†^	SS^†^, N (%)	35 (89.7)	36 (90.0)	38 (85.7)	41 (83.7)	0.799^a^

*RIF = Rifampicin; ALB = Albendazole; TA = Treatment Arm; SS = Skin Snips; N = Number; CI = Confidence interval; PP = per-protocol*

*^†^* *Only snips with at least 1 MF count and a positive OvActin signal were used for analysis.*

^*a*^ *Fisher’s Exact test calculated using Stata version 16*

The qPCR results indicated no statistical differences in OvActin copy numbers across treatment arms (p > 0.05) when MF DNA was amplified from skin snips obtained 4-month post-treatment.

**Fig 2:**
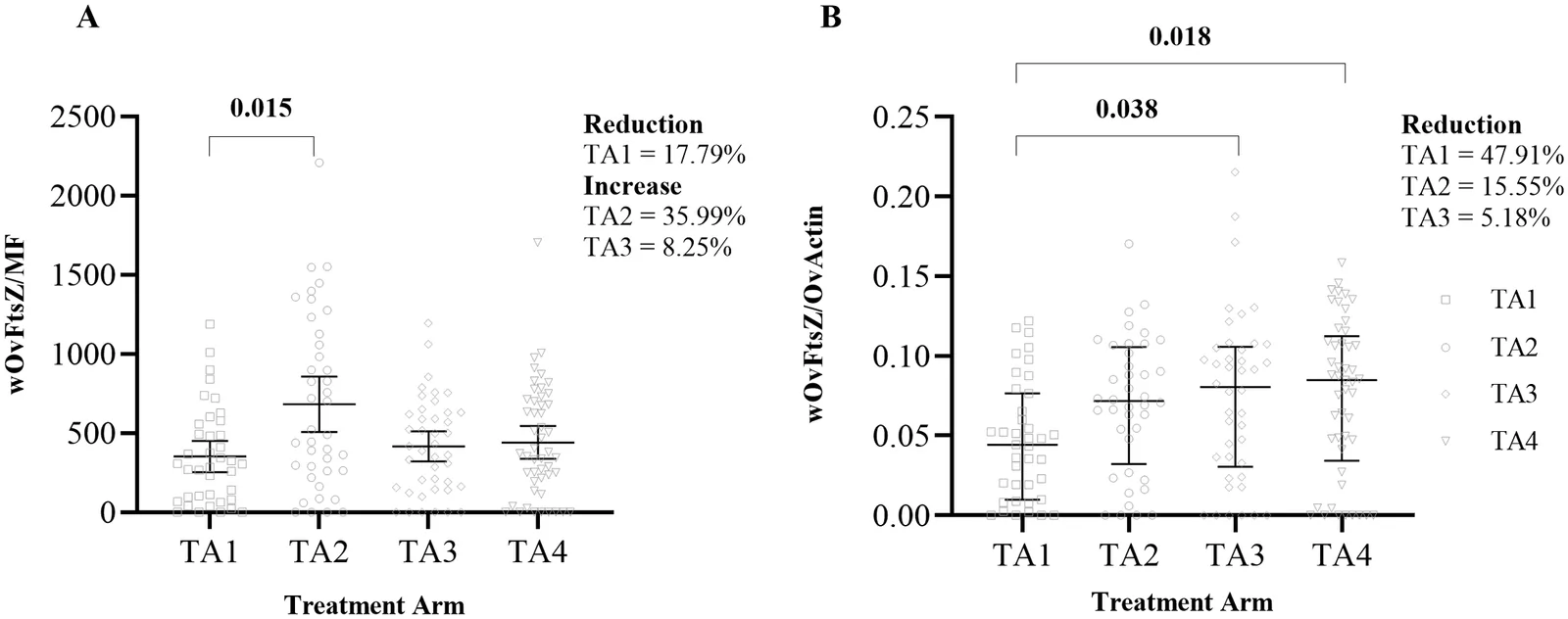
Effects of treatment against *Wolbachia* in skin microfilariae—4-months post-treatment—PP analysis. *TA = Treatment arm; PP = per-protocol; TA1 = RIF + ALB – 14 days; TA2 = RIF + ALB – 7 days; TA3 = ALB alone – 14 days; TA4 = No treatment/control. Graphs show the median values and interquartile ranges. Kruskal-Wallis with Dunn’s multiple comparison tests, with Holm-Bonferroni correction applied, calculated using Stata version 16. The following p-values indicate statistically significant differences before correction, but did not remain significant after correction: wOvFtsZ/MF: TA2 vs TA3, p = 0.026, TA1 vs TA4, p = 0.029; wOvFtsZ/OvActin: TA2 vs TA1, p = 0.014*.

Changes in *Wolbachia* levels post-treatment were calculated as percentage median reductions relative to the no treatment control. No differences in wOvFtsZ/MF ratios were observed between the treated and the control arms (Figs 2A and S4A). However, a reduction in this index was observed between the RIF + ALB – 7 Days (TA2) and RIF + ALB – 14 Days (TA1) (PP: p = 0.015), with TA1 showing a 17.79% reduction.

The wOvFtsZ/OvActin ratio revealed a more pronounced treatment effect. A median reduction in *Wolbachia* load was observed in the RIF + ALB – 14 Days (TA1) at a rate of 47.91%, compared to the No Treatment/Control group (TA4) (PP: p = 0.018). All other treatments were less effective than the 14 Days RIF + ALB regimen in reducing *Wolbachia* loads (p > 0.05) (Figs 2B and S4B). Additionally, RIF + ALB – 14 Days (TA1) also had lower wOvFtsZ/OvActin *Wolbachia* loads compared to ALB alone – 14 Days (TA3) (PP: p = 0.022), indicating superior efficacy of the 14 Days RIF + ALB combination over ALB alone – 14 Days monotherapy (Figs 2A and S4A), potentially indicating the action of RF.

### *Wolbachia* depletion from nodules sections

Real-time qPCR was used to support immunohistological findings by quantitatively assessing *Wolbachia* depletion in worms in nodule tissue sections (S18 Table). *O. volvulus* DNA was extracted from 8 consecutive sections per nodule, adjacent to sections used for histology to maximise DNA yield and analysed for *Wolbachia* FtsZ and *O. volvulus* actin. Out of the 196 nodules evaluated by immunohistology, DNA was successfully isolated from 152 nodules. The remaining nodules were calcified and had been treated with EDTA for immunohistology, making DNA extraction from them not possible. Due to variations in the amount of human tissue relative to worms, and between nodules, *Wolbachia* loads were normalised to *O. volvulus* actin levels following the method described by Gilbert *et al.*, (2005) [51]. Nodules containing only dead worms, confirmed by histology or lacking detectable actin signals were excluded from further analysis (PP: n = 20; ITT: n = 22). The absence of an actin signal likely indicates absence of worms in the nodule section.

The qPCR results (S18–S20 Tables) generally corroborated the immunohistology findings, showing no differences in *Wolbachia* depletion in most treatment arms, compared to the control. Normalised median *Wolbachia* loads (wOvFtsZ/OvActin) increased by 42.69% in RIF + ALB – 14 Days (TA1) and 28.65% in RIF + ALB – 7 Days (TA2), while ALB alone – 14 Days (TA3) showed a reduction of 21.05%. However, these changes were not significant (p > 0.05).

### Changes in microfilarial densities following treatment

Microfilarial density, measured as MF/mg skin snip, serves as the standard measure of infection intensity. At baseline, no differences in the microfilarial loads (MF/mg skin snip) were observed across all treatment arms (p = 0.678). At 20-months post-treatment, the highest microfilarial density was observed in the No Treatment/Control group (TA4), while the lowest was recorded in ALB alone –14 days (TA3). Despite the observed variations, there were no statistical differences in microfilaridermia between all arms at 4-, 18- and 20-months post-treatment assessment (Table 3).

**Table 3:** Changes in microfilarial densities 4-, 18-, and 20- months following treatment—PP analysis.

		RIF + ALB – 14 Days (TA1)	RIF + ALB – 7 Days (TA2)	ALB alone – 14 Days (TA3)	No Treatment/ Control (TA4)	P-value
**Baseline**						
Participants	N	29	30	29	30	
MF Positive	N (%)	29 (100)	30 (100)	29 (100)	30 (100)	
MF/mg skin snip	Median	0.75	0.83	0.73	0.77	0.678^a^
	Min-Max	0.05-15.75	0.08-15.81	0.10-64.73	0.06-34.71	
	Percentiles 25^th^- 75^th^	0.33;1.17	0.33;2.87	0.36;3.13	0.13;4.85	
	GM	0.63	0.91	1.12	0.76	
	95% CI of the GM	0.36-1.12	0.55-1.51	0.62-2.04	0.44-1.30	
**4 Months** ^**¥**^						
Participants	N	29	28	28	28	
MF Positive	N (%)	22 (75.86)	23 (82.14)	24 (85.71)	27 (96.43)	0.167^b^
MF/mg skin snip	Median	0.53	0.51	0.94	1.53	0.228^a^
	Min-Max	0.00-66.28	0.00-6.26	0.00-52.46	0.00-33.77	
	Percentiles 25^th^- 75^th^	0.16;1.81	0.20;1.65	0.13;1.20	0.23;3.56	
	GM	0.12	0.14	0.26	0.90	
	95% CI of the GM	0.02-0.59	0.03-0.60	0.06-1.03	0.33-2.42	
	P-value*	0.996	0.150	0.018	0.034	
**18 Months** ^**ψ**^						
Participants	N	22	25	25	24	
MF Positive	N (%)	16 (72.73)	20 (80.00)	15 (60.00)	19 (79.17)	0.385^b^
MF/mg skin snip	Median	0.87	0.46	0.07	0.53	0.172^a^
	Min-Max	0.00-17.20	0.00-16.13	0.00-19.17	0.00-11.92	
	Percentiles 25^th^- 75^th^	0.00;2.67	0.17;1.43	0.00;0.72	0.10;2.41	
	GM	0.11	0.14	0.02	0.14	
	95% CI of the GM	0.01-0.79	0.03-0.69	0.00-0.11	0.03-0.79	
	P-value*	0.610	0.071	0.007	0.726	
**20 Months** ^₼^						
Participants	N	21	25	25	23	
MF Positive	N (%)	16 (76.00)	21 (84.00)	16 (64.00)	19 (82.61)	0.369^b^
MF/mg skin snip	Median	0.86	1.25	0.15	1.6	0.087^a^
	Min-Max	0.00-34.90	0.00-12.18	0.00-20.56	0.00-26.98	
	Percentiles 25^th^- 75^th^	0.13;3.02	0.20;3.22	0.00;0.59	0.06;6.11	
	GM	0.15	0.33	0.03	0.32	
	95% CI of the GM	0.02-1.06	0.07-1.60	0.00-0.18	0.05-1.90	
	P-value*	0.973	0.812	0.003	0.151	

*RIF = Rifampicin; ALB = Albendazole; TA = Treatment arm; N = Number; GM = Geometric mean; CI = Confidence interval; PP = per-protocol.*

^*a*^ *Kruskal-Wallis test.*

^*b*^ *Fisher Exact test.*

*Comparison to baseline (Wilcoxon signed-rank test)

*Geometric means adjusted by adding 0.0001 to the MF/mg skin snip values to account for zero values.*

*^**¥**^* *Participants who were treated per protocol (PP) and present at 4 months post-treatment*

*^**ψ**^* *Participants who were treated per protocol (PP) and present at 18 months post treatment, and had taken IVM at 6 months were included in the analysis*

₼*Participants who were treated per protocol (PP) and present at 20 months post treatment, and had taken IVM at 6 months were included in the analysis*

*Statistics calculated using Stata version 16*

Longitudinally, a slight increase in skin MF/mg skin snip was observed 4 months post-treatment compared to baseline in the ALB alone– 14 days group (TA3) (p = 0.018) (Table 3). However, reductions were observed at 18- (p = 0.007) and 20-months (p = 0.003), indicating a delayed but substantial decline in microfilarial densities following treatment (Table 3). Similarly, an increase in MF/mg skin snip was detected in the No Treatment/Control group (TA4) at 4 months in the PP analysis (p = 0.034; Table 3). However, this increase was not significant in the ITT analysis (S22 Table) and was not sustained at the 18- and 20-month time points, indicating that the rise at 4-months was transient.

## Discussion

This study represents the first clinical evaluation of high-dose Rifampicin (RIF) combined with Albendazole (ALB) as an alternative regimen for onchocerciasis in humans. This is in response to the failure of IVM in clearing MF in areas with decades of the treatment, potentially due to IVM resistance or sub-optimal parasite clearance [52,53]. Potential adulticidal drugs such as high-dose RIF plus ALB targets adult worm embryogenesis, leading to reduction or clearance of skin MF, aiming to complement efforts to achieving the WHO 2030 elimination target [4].

In this current trial, the safety profile of high-dose RIF in combination with ALB, was favourable. Adverse events were generally mild and included expected effects such as reddish urine discoloration, headache, and transient fever. Temporary elevations in liver enzymes were also observed but resolved spontaneously, consistent with previous pharmacokinetic and pharmacodynamic studies on the safety of short-course, high-dose RIF [12,34]. Concerns regarding resistance development, particularly given RIF’s role in tuberculosis treatment, are mitigated by the short duration of administration for filarial indications and the absence of evidence linking short-term use to clinically significant resistance [12]. It should be noted that no pharmacokinetic bioanalysis was performed; therefore, steady-state drug exposure and potential drug–drug interactions could not be confirmed and remain speculative in the context of this study.

In evaluating the macrofilaricidal efficacy of the combination therapy (high-dose RIF plus ALB), we did not observe a statistically significant increase in adult worm mortality or inhibition of embryogenesis, compared to the control arm. These outcomes contrast with preclinical studies that demonstrated significant *Wolbachia* depletion and partial reductions in female worm burden with similar regimens [12,25], and are likewise inconsistent with the findings of our lymphatic filariasis (LF) trial, using the same regimen [54]. In our LF clinical trial, we observed an early, sustained and significant reductions in circulating filarial antigen (CFA) levels in the RIF plus ALB groups even though there was no consistent and sustained decline in CFA negativity across time points beyond 4 months [54].

The limited efficacy observed in the current study may be attributed to several factors. RIF, despite achieving high tissue concentrations, has a short half-life (2–5 hours) which may necessitate frequent dosing to maintain therapeutic levels [55], partly informing the rationale for administering the regimen for a minimum of 7 days. ALB absorption is variable and influenced by dietary fat intake [56]; therefore, participants were instructed to eat prior to their daily treatment dose. It is acknowledged that food intake enhances ALB absorption [57,58], and RIF is generally recommended to be taken on an empty stomach [32,59]. However, co-administration of RIF and ALB was done in this study to reduce the tendency of stomach upsets or nausea; a choice that reflects the real-world administration conditions anticipated for its potential use as an MDA drug [60,61]. Although pharmacokinetic assessments were not conducted, we considered that variability related to food intake would have minimal impact on drug bioavailability, as treatments were administered within participants’ communities around mid‑morning. Nevertheless, because the interval between food consumption and treatment dosing was not recorded, RIF bioavailability may have been affected in participants who ate within 2 hours prior to drug administration.

It should be noted that the same treatment modalities were applied in our LF trial [54], which demonstrated marked efficacy of the drug regimen against *W. bancrofti* infection. The contrasting outcomes between these two trials more likely reflect biological differences between *O. volvulus* and *W. bancrofti*. Nodular tissue compartmentalisation, greater worm biomass, and potentially differing *Wolbachia* susceptibility thresholds in *O. volvulus* may serve as barriers to achieving comparable macrofilaricidal effects. Importantly, Turner *et al.* (2017) reported that RIF did not adversely affect ALB bioavailability under controlled conditions; however, their study was conducted in mice [25]. Human-specific metabolic pathways may differ from those in preclinical models, and interindividual variation in drug metabolism, including variation across different ethnic backgrounds, may further influence systemic drug exposure in clinical settings [62,63]. Since no pharmacokinetic assessment was conducted in this trial, the systemic exposure levels of both drugs remain unknown and can only be assumed to have been near optimal.

Systemic drug distribution and nodule vascularisation in humans may also differ from preclinical models, potentially limiting drug penetration and reducing effective concentrations at target sites. The ‘nodular sanctuary effect’, in which dense fibrous tissue in onchocerciasis nodules limits drug access, is a recognised barrier to achieving significant macrofilaricidal activity [64–66], and may have contributed to insufficient intranodular drug concentrations, thereby failing to disrupt female worm reproductive functions effectively. It is also important to acknowledge that the preclinical model used to define the RIF regimen evaluated in this trial may not fully represent the biological and pharmacological complexity of adult *O. volvulus* infections in humans. The male *O. ochengi* implant model in SCID mice [12] differs from the human clinical setting in parasite species, worm biomass, parasite location, and nodular tissue architecture. Indeed, no reliable small animal model of microfilarial *O. volvulus* infection currently exists [67], which limits the accuracy of pharmacokinetic-pharmacodynamics predictions derived from this surrogate model [2]. This translational gap highlights the need for empirical dose-ranging studies in humans that incorporate pharmacokinetic assessment to establish the drug exposure levels required for effective anti-*Wolbachia* activity in the biological context of human onchocerciasis. Studies utilising longer treatment durations (4–6 weeks) with alternative anti-*Wolbachia* agents, such as DOX alone or in combination with ALB, have demonstrated superior effects on adult worm viability and embryogenesis [16,68].

Male worms exhibited consistently lower mortality rates across all treatment arms, likely due to their reduced dependence on *Wolbachia* for survival, a finding consistent with prior studies [25,36]. Female worms, which rely heavily on *Wolbachia* for reproduction and survival, showed higher mortality rates, though the observed effects were modest, compared to preclinical outcomes.

Regarding RIF’s potential to deplete *Wolbachia* in *O. volvulus*, our findings present a complex picture. At 4 months post-treatment, the 14-day RIF + ALB regimen achieved a statistically significant 47.9% reduction in *Wolbachia* load in skin MF as determined by qPCR. Although this was lower than the > 90% reductions reported in preclinical studies using high-dose RIF in adult worms of mouse models [12,25], *Wolbachia* depletion in MF nevertheless provides a useful surrogate for adult worm histology, and indicates measurable *anti-Wolbachia* activity in humans. It is noteworthy, however that skin MF are dermally resident and therefore directly accessible to systemically circulating drug levels, without the physical barrier of fibrous nodular tissue. The sub-optimal *Wolbachia* depletion in skin MF observed at 4 months may be suggestive of lower than expected systemic RIF exposure levels required for achieving >90% *Wolbachia* depletion, as defined by Aljayyoussi *et al.* (2017) [12]. However, this observation is inconsistent with earlier DOX studies in onchocerciasis and LF that showed >90% *Wolbachia* depletion necessary for a sustained anti-*Wolbachia* effect [11,47,69]. By 20 months post-treatment, immunohistological evaluation of extirpated nodules revealed no significant *Wolbachia* depletion in adult worms across treatment arms relative to the No Treatment/Control arm. This finding is consistent with recent experimental evidence showing that *Wolbachia* can rebound to baseline levels within months of antibiotic treatment in preclinical models [70]. One proposed mechanism for this recrudescence involves *Wolbachia* populations sheltered within ovarian sheath cells that may be less accessible to antibiotic penetration [71]. While this remains a plausible explanation, the hypothesis is still preliminary, as prior anti-*Wolbachia* regimens using DOX achieved >90% depletion [16], an efficacy anticipated from RIF plus ALB combination therapy. The contrasting outcomes between skin MF and adult worms likely reflect differences in parasite stage, tissue compartmentalisation, and drug pharmacokinetics. While RIF and ALB may reach skin microfilariae effectively, drug penetration into fibrous nodules housing adult worms remains a key unresolved challenge [65,66]. Moreover, the 14-day treatment duration may have been insufficient to sustain therapeutic drug levels long enough for durable *Wolbachia* depletion, particularly within nodules [72]. Collectively these findings underscore the need to reassess dosing strategies and to explore formulations or delivery systems that enhance drug retention and tissue penetration.

To improve the therapeutic potential of RIF + ALB, adjustments to dosing regimens may be required. Extending RIF treatment beyond 14 days, and as suggested by Specht *et al.* (2008), combinatory therapy other than ALB such as oxfendazole which has demonstrated good synergistic effects with other drugs could allow for a more sustained *Wolbachia* depletion and increase the likelihood of worm death [35]. Likewise, other formulations with enhanced bioavailability and tissue penetration such as benzimidazole may strengthen the synergistic anti-*Wolbachia* effect when combined with RIF [73].

Regarding the effects of the study medications on skin microfilarial loads, baseline microfilarial densities were comparable across all arms. It is important to note that neither RIF nor ALB exerts direct microfilaricidal activity. RIF acts indirectly by depleting *Wolbachia* endosymbionts within adult female worms, which subsequently disrupts embryogenesis and leads to gradual decline in MF production over time [12,25]. ALB and other benzimidazole similarly act indirectly, primarily by inhibiting β-tubulin polymerisation in nematodes, which disrupts microtubule formation and interferes with the female embryogenesis pathway, rather than directly killing existing MF in the skin [22,74]. Meaningful reductions in skin microfilarial loads from either drugs are therefore dependent on the extent and durability of their effects on adult worm reproductive function [16]. In the ALB alone (14-day) group, significant reductions were observed at 18- and 20-months post-treatment, though not at 4-months, where a slight but significant increase was seen, suggesting a delayed but substantial response to ALB treatment over time. The combination regimens did not achieve reductions comparable to those reported for ivermectin, which has been shown to deplete over 99% of MF within 1–2 months after administration [75,76], and differences between the RIF plus ALB arms and the No Treatment arm differences were not statistically significant. The limited efficacy of the combination regimens is consistent with Richards *et al.*, (2007), who found that RIF-based regimens did not significantly reduce microfilarial loads at 9 months post-treatment [36], reinforcing the broader principle that antibiotics alone do not rapidly clear MF because they act indirectly through *Wolbachia* depletion rather than through direct microfilaricidal activity [16]. Meaningful early reductions in MF are therefore more likely when antibiotics are combined with a direct microfilaricidal agent such as IVM, as demonstrated in previous DOX plus IVM combination studies [16,77].

The reductions in microfilarial densities observed in the ALB alone (14-day) treatment arm reaffirm its potential as a standalone therapy in specific contexts [37]. Improved drug bioavailability and host immune responses, in the absence of pharmacokinetic interference from RIF, likely contributed to this outcome. RIF is a potent inducer of cytochrome P450 enzymes and may accelerates ALB biotransformation, thereby reducing systemic ALB exposure and potentially diminishing efficacy during co-administration [74,78], an interaction that may have undermined the combined effects of RIF + ALB in this trial.

The increase in microfilarial densities observed in TA4 (No treatment) at 4-months is consistent with the natural dynamics of *O. volvulus*, where adult worms continuously produce MF in the absence of intervention. The observed fluctuations in microfilarial counts in this arm across timepoints likely reflect the interplay of ongoing parasite reproduction, seasonal transmission dynamics [79], and host immune modulation [80], rather than any treatment effect. While similar patterns of stable or increasing microfilarial loads in untreated individuals have been reported in IVM-naïve populations in Cameroon [53,81,82], it is worth noting that the IVM dose administered at the 6-months follow-up visit may also have contributed to the variability observed in microfilarial counts at subsequent timepoints in the No treatment arm. It is also notable that the significant increase in the no-treatment arm was observed only in the PP analysis and not in the ITT analysis, suggesting this finding should be interpreted with caution.

Anti-*Wolbachia* therapy has been validated as a macrofilaricidal approach across filarial infections, but important differences exist between *O. volvulus* and the lymphatic filariae. In LF, the same high-dose RIF plus ALB regimen produced evidence of macrofilaricidal activity against *W. bancrofti* in our Ghana trial [54]. By contrast, earlier field studies in onchocerciasis found no significant macro- or microfilaricidal effects from short-course RIF regimens [36], and even 4-week RIF treatment only modestly outperformed shorter courses [35]. DOX has shown more consistent macrofilaricidal and sterilising effects across multiple filarial systems, including *O. volvulus* [16,68], *W. bancrofti* [83,84], and *B. malayi* [85], though requiring 4–6 weeks of treatment. Taken together, these observations indicate that while LF species such as *W. bancrofti* and *Brugia* respond robustly to short-course anti-*Wolbachia* regimens, *O. volvulus* appear more refractory, requiring longer or more intensive antibiotic exposure to achieve sustained *Wolbachia* depletion. This highlights the need for new short-course macrofilaricidal candidates.

A key limitation of our study was the inability to assess *Wolbachia* depletion in skin MFs at 18-and 20-months post-treatment, primarily due to resource constraints. Although sustained *Wolbachia* depletion was not observed in adult worms at 20 months, corresponding longitudinal data from MFs would have enabled direct comparison across parasite life stages, assessment of potential bacterial recrudescence, and deeper insights into the temporal dynamics of *Wolbachia* depletion. The absence of these measurements therefore limited a more comprehensive evaluation of long-term treatment effects. As discussed earlier, the lack of pharmacokinetic assessment means it cannot be confirmed whether the target steady-state RIF exposure was achieved in trial participants, and the absence of RIF monotherapy arms precludes evaluation of whether drug-drug interactions between RIF and ALB may have adversely influenced treatment outcomes. Together, these represent significant limitations that hamper definitive conclusions about the intrinsic efficacy of the RIF plus ALB regimen for onchocerciasis. Nonetheless, the study provides important insights into the short-term effects of high-dose RIF combined with ALB and highlights the need for strategies capable of achieving more durable therapeutic efficacy.

In conclusion, this study confirms the safety of high-dose RIF in combination with ALB for treating onchocerciasis. The limited macrofilaricidal effects observed suggest that treatment duration and pharmacokinetic properties were insufficient to achieve sustained *Wolbachia* depletion, and subsequent adult worm death or inhibition of embryogenesis. These findings highlight the need for longer or optimised combination regimens, as well as incorporation of pharmacokinetic assessments in future trials to verify target drug exposure levels. The reductions in microfilarial densities observed in the ALB alone (14 days) treatment arm reaffirm its potential as a standalone therapy in specific contexts. Translating the preclinical success of RIF plus ALB combination therapy into clinical efficacy for onchocerciasis remains a challenge, and future studies should evaluate whether extended RIF courses, alternative combination regimens, or as improved benzimidazole formulations with enhanced bioavailability and tissue penetration, can strengthen anti-*Wolbachia* effects and complement efforts towards onchocerciasis elimination.

## Supporting information

S1 Fig qPCR DNA quantification standard curve for gBlock (OvActin) at a 10-fold dilution. (TIF)

S2 Fig Participant flow chart showing treatment allocation, presence/absence during follow-up visits, data analysis set. ITT and/or PP analysis sets, and reason for absence or exclusion from PP analysis.
(TIF)

S3 Fig Adverse Events experienced by participants. (TIF)

S4 Fig Effects of study drugs against *Wolbachia in* skin microfilariae at 4 months post-treatment—ITT analysis. (TIF)

S5 Fig Changes in microfilarial densities 4-, 18-, and 20- months following treatment—PP analysis. (TIF)

S6 Fig Changes in microfilarial densities 4-, 18-, and 20- months following treatment—ITT analysis. (TIF)

S1 Table Primers, Probes and Standards (gBlocks) sequences used for the real-time qPCR. (DOCX)

S2 Table Participant compliance with treatment regimens. (DOCX)

S3 Table Adverse event assessment. (DOCX)

S4 Table Adverse event assessment, detailed. (DOCX)

S5 Table Effect of treatment on adult worm—PP analysis. (DOCX)

S6 Table Effect of treatment on adult worms—ITT analysis. (DOCX)

S7 Table Effect of treatment on embryogenesis—PP analysis. (DOCX)

S8 Table Effect of treatment on embryogenesis: statistics^a,b^—PP analysis. (DOCX)

S9 Table Effect of treatment on embryogenesis—ITT analysis. (DOCX)

S10 Table Effect of treatment on embryogenesis: statistics^a,b^–ITT analysis. (DOCX)

S11 Table Effect of treatment on intranodular microfilariae—PP analysis. (DOCX)

S12 Table Effect of treatment on intranodular microfilariae (MF)—ITT analysis. (DOCX)

S13 Table Effect of treatment on the presence of *Wolbachia* in female worms: immunohistology—PP analysis. (DOCX)

S14 Table Effect of treatment on the presence of *Wolbachia* in female worms: immunohistology—ITT analysis. (DOCX)

S15 Table Effect of treatment on the presence of *Wolbachia* in male worms: immunohistology—PP analysis. (DOCX)

S16 Table Effect of treatment on the presence of *Wolbachia* in male worms: immunohistology—ITT analysis. (DOCX)

S17 Table Selection of skin snip samples for qPCR and *Wolbachia* depletion analysis—ITT analysis. (DOCX)

S18 Table Effect of treatment on the presence of *Wolbachia* in nodule sections: qPCR^a^—PP analysis. (DOCX)

S19 Table Effect of treatment on the presence of *Wolbachia* in nodule sections: statistics^a,b^—PP analysis. (DOCX)

S20 Table Effect of treatment on the presence of *Wolbachia* in nodule sections: PCR^a^—ITT analysis. (DOCX)

S21 Table Effect of treatment on the presence of *Wolbachia* in nodule sections: statistics^a,b^—ITT analysis. (DOCX)

S22 Table Changes in microfilarial densities 4-, 18-, and 20- months following treatment —ITT analysis. (DOCX)

S23 Table Iron deposition in adult worm—PP analysis. (DOCX)

S24 Table Iron deposition in adult worm—ITT analysis. (DOCX)

S25 Table Viability status of adult worm —PP analysis. (DOCX)

S26 Table Viability status of adult worm—ITT analysis. (DOCX)

S27 Table Consort check list. (DOCX)

## References

[1] WHO. Onchocerciasis: Key facts. World Health Organization. 2022 [cited 2022 December 19]. Available from: https://www.who.int/news-room/fact-sheets/detail/onchocerciasis

[2] Ngwewondo A, Scandale I, Specht S. Onchocerciasis drug development: from preclinical models to humans. Parasitol Res. 2021;120(12):3939–64. doi: 10.1007/s00436-021-07307-4 34642800 PMC8599318

[3] Ubachukwu PO. Socio-Economic Impact of Onchocerciasis with Particular Reference to Females and Children: A Review. Anim Res Int. 2008;3:494–504. doi: 10.4314/ari.v3i2.40778

[4] WHO. Onchocerciasis: key facts. World Health Organization. 2025 [cited 2025 June 6]. Available from: https://www.who.int/news-room/fact-sheets/detail/onchocerciasis

[5] Traore MO, Sarr MD, Badji A, Bissan Y, Diawara L, Doumbia K, et al. Proof-of-principle of onchocerciasis elimination with ivermectin treatment in endemic foci in Africa: final results of a study in Mali and Senegal. PLoS Negl Trop Dis. 2012;6(9):e1825. doi: 10.1371/journal.pntd.0001825 23029586 PMC3441490

[6] Zarroug IMA, Hashim K, ElMubark WA, Shumo ZAI, Salih KAM, ElNojomi NAA, et al. The First Confirmed Elimination of an Onchocerciasis Focus in Africa: Abu Hamed, Sudan. Am J Trop Med Hyg. 2016;95(5):1037–40. doi: 10.4269/ajtmh.16-0274 27352878 PMC5094213

[7] Osei-Atweneboana MY, Awadzi K, Attah SK, Boakye DA, Gyapong JO, Prichard RK. Phenotypic evidence of emerging Ivermectin resistance in Onchocerca volvulus. PLoS Negl Trop Dis. 2011;5(3):e998. doi: 10.1371/journal.pntd.0000998 21468315 PMC3066159

[8] Makenga Bof J-C, Maketa V, Bakajika DK, Ntumba F, Mpunga D, Murdoch ME, et al. Onchocerciasis control in the Democratic Republic of Congo (DRC): challenges in a post-war environment. Trop Med Int Health. 2015;20(1):48–62. doi: 10.1111/tmi.12397 25302560

[9] Wanji S, Kengne-Ouafo JA, Esum ME, Chounna PWN, Adzemye BF, Eyong JEE, et al. Relationship between oral declaration on adherence to ivermectin treatment and parasitological indicators of onchocerciasis in an area of persistent transmission despite a decade of mass drug administration in Cameroon. Parasit Vectors. 2015;8:667. doi: 10.1186/s13071-015-1283-6 26715524 PMC4696282

[10] Boussinesq M, Gardon J, Gardon-Wendel N, Chippaux J-P. Clinical picture, epidemiology and outcome of Loa-associated serious adverse events related to mass ivermectin treatment of onchocerciasis in Cameroon. Filaria J. 2003;2 Suppl 1(Suppl 1):S4. doi: 10.1186/1475-2883-2-S1-S4 14975061 PMC2147657

[11] Turner JD, Tendongfor N, Esum M, Johnston KL, Langley RS, Ford L, et al. Macrofilaricidal activity after doxycycline only treatment of Onchocerca volvulus in an area of Loa loa co-endemicity: a randomized controlled trial. PLoS Negl Trop Dis. 2010;4(4):e660. doi: 10.1371/journal.pntd.0000660 20405054 PMC2854122

[12] Aljayyoussi G, Tyrer HE, Ford L, Sjoberg H, Pionnier N, Waterhouse D, et al. Short-Course, High-Dose Rifampicin Achieves Wolbachia Depletion Predictive of Curative Outcomes in Preclinical Models of Lymphatic Filariasis and Onchocerciasis. Sci Rep. 2017;7(1):210. doi: 10.1038/s41598-017-00322-5 28303006 PMC5428297

[13] Turner JD, Marriott AE, Hong D, O’ Neill P, Ward SA, Taylor MJ. Novel anti-Wolbachia drugs, a new approach in the treatment and prevention of veterinary filariasis?. Vet Parasitol. 2020;279:109057. doi: 10.1016/j.vetpar.2020.109057 32126342

[14] Chirgwin SR, Coleman SU, Porthouse KH, Nowling JM, Punkosdy GA, Klei TR. Removal of Wolbachia from Brugia pahangi is closely linked to worm death and fecundity but does not result in altered lymphatic lesion formation in Mongolian gerbils (Meriones unguiculatus). Infect Immun. 2003;71(12):6986–94. doi: 10.1128/IAI.71.12.6986-6994.2003 14638788 PMC308881

[15] Sharma R, Al Jayoussi G, Tyrer HE, Gamble J, Hayward L, Guimaraes AF, et al. Minocycline as a re-purposed anti-Wolbachia macrofilaricide: superiority compared with doxycycline regimens in a murine infection model of human lymphatic filariasis. Sci Rep. 2016;6:23458. doi: 10.1038/srep23458 26996237 PMC4800446

[16] Debrah AY, Specht S, Klarmann-Schulz U, Batsa L, Mand S, Marfo-Debrekyei Y, et al. Doxycycline Leads to Sterility and Enhanced Killing of Female Onchocerca volvulus Worms in an Area With Persistent Microfilaridermia After Repeated Ivermectin Treatment: A Randomized, Placebo-Controlled, Double-Blind Trial. Clin Infect Dis. 2015;61(4):517–26. doi: 10.1093/cid/civ363 25948064 PMC4518165

[17] Landmann F, Voronin D, Sullivan W, Taylor MJ. Anti-filarial activity of antibiotic therapy is due to extensive apoptosis after Wolbachia depletion from filarial nematodes. PLoS Pathog. 2011;7(11):e1002351. doi: 10.1371/journal.ppat.1002351 22072969 PMC3207916

[18] Hoerauf A, Specht S, Büttner M, Pfarr K, Mand S, Fimmers R, et al. Wolbachia endobacteria depletion by doxycycline as antifilarial therapy has macrofilaricidal activity in onchocerciasis: a randomized placebo-controlled study. Med Microbiol Immunol. 2008;197(3):295–311. doi: 10.1007/s00430-007-0062-1 17999080 PMC2668626

[19] Hoerauf A, Specht S, Marfo-Debrekyei Y, Büttner M, Debrah AY, Mand S, et al. Efficacy of 5-week doxycycline treatment on adult Onchocerca volvulus. Parasitol Res. 2009;104(2):437–47. doi: 10.1007/s00436-008-1217-8 18850111

[20] Taylor MJ, Hoerauf A, Townson S, Slatko BE, Ward SA. Anti-Wolbachia drug discovery and development: safe macrofilaricides for onchocerciasis and lymphatic filariasis. Parasitology. 2014;141(1):119–27. doi: 10.1017/S0031182013001108 23866958 PMC3884836

[21] Walker M, Specht S, Churcher TS, Hoerauf A, Taylor MJ, Basáñez M. Therapeutic Ef fi cacy and Macro fi laricidal Activity of Doxycycline for the Treatment of River Blindness. 2015;60(8):1199–1207.doi: 10.1093/cid/ciu1152 25537873 PMC4370165

[22] Serbus LR, Landmann F, Bray WM, White PM, Ruybal J, Lokey RS, et al. A cell-based screen reveals that the albendazole metabolite, albendazole sulfone, targets Wolbachia. PLoS Pathog. 2012;8(9):e1002922. doi: 10.1371/journal.ppat.1002922 23028321 PMC3447747

[23] Sharma R, Hoti SL, Vasuki V, Sankari T, Meena RL, Das PK. Filamentation temperature-sensitive protein Z (FtsZ) of Wolbachia, endosymbiont of Wuchereria bancrofti: a potential target for anti-filarial chemotherapy. Acta Trop. 2013;125(3):330–8. doi: 10.1016/j.actatropica.2012.12.004 23262214

[24] Li Z, Garner AL, Gloeckner C, Janda KD, Carlow CK. Targeting the Wolbachia cell division protein FtsZ as a new approach for antifilarial therapy. PLoS Negl Trop Dis. 2011;5(11):e1411. doi: 10.1371/journal.pntd.0001411 22140592 PMC3226453

[25] Turner JD, Sharma R, Al Jayoussi G, Tyrer HE, Gamble J, Hayward L, et al. Albendazole and antibiotics synergize to deliver short-course anti-Wolbachia curative treatments in preclinical models of filariasis. Proc Natl Acad Sci U S A. 2017;114(45):E9712–21. doi: 10.1073/pnas.1710845114 29078351 PMC5692564

[26] Boeree MJ, Heinrich N, Aarnoutse R, Diacon AH, Dawson R, Rehal S, et al. High-dose rifampicin, moxifloxacin, and SQ109 for treating tuberculosis: a multi-arm, multi-stage randomised controlled trial. Lancet Infect Dis. 2017;17(1):39–49. doi: 10.1016/S1473-3099(16)30274-2 28100438 PMC5159618

[27] SADA. Sefwi district medium term development plan-2018-2021. 2017.

[28] Hamley JID, Blok DJ, Walker M, Milton P, Hopkins AD, Hamill LC, et al. What does the COVID-19 pandemic mean for the next decade of onchocerciasis control and elimination?. Trans R Soc Trop Med Hyg. 2021;115(3):269–80. doi: 10.1093/trstmh/traa193 33515042 PMC7928565

[29] WHO. Onchocerciasis key facts. World Health Organization. 2019 [cited 2021 June 6]. Available from: https://www.who.int/news-room/fact-sheets/detail/onchocerciasis

[30] Julious SA. Sample size of 12 per group rule of thumb for a pilot study. Pharm Stat. 2005;4:287–91. doi: 10.1002/pst.185

[31] Médecins Sans Frontières (MSF). Rifampicin (R). Medical Guidelines. 2026 [cited 2026 April 24]. Available from: https://medicalguidelines.msf.org/en/viewport/TUB/english/rifampicin-r-20323858.html

[32] Peloquin CA, Namdar R, Singleton MD, Nix DE. Pharmacokinetics of rifampin under fasting conditions, with food, and with antacids. Chest. 1999;115(1):12–8. doi: 10.1378/chest.115.1.12 9925057

[33] Zent C, Smith P. Study of the effect of concomitant food on the bioavailability of rifampicin, isoniazid and pyrazinamide. Tuber Lung Dis. 1995;76(2):109–13. doi: 10.1016/0962-8479(95)90551-0 7780091

[34] Wan Sulaiman WA, Kamtchum-Tatuene J, Mohamed MH, Ramachandran V, Ching SM, Sazlly Lim SM, et al. Anti-Wolbachia therapy for onchocerciasis & lymphatic filariasis: Current perspectives. Indian J Med Res. 2019;149(6):706–14. doi: 10.4103/ijmr.IJMR_454_17 31496523 PMC6755775

[35] Specht S, Mand S, Marfo-Debrekyei Y, Debrah AY, Konadu P, Adjei O, et al. Efficacy of 2- and 4-week rifampicin treatment on the Wolbachia of Onchocerca volvulus. Parasitol Res. 2008;103(6):1303–9. doi: 10.1007/s00436-008-1133-y 18679718

[36] Richards FO Jr, Amann J, Arana B, Punkosdy G, Klein R, Blanco C, et al. No depletion of Wolbachia from Onchocerca volvulus after a short course of rifampin and/or azithromycin. Am J Trop Med Hyg. 2007;77(5):878–82. doi: 10.4269/ajtmh.2007.77.878 17984346

[37] Cline BL, Hernandez JL, Mather FJ, Bartholomew R, Maza SND, Rodulfo S. Albendazole in the treatment of onchocerciasis: double-blind clinical trial in Venezuela. Am J Trop Med Hyg. 1992;47(3):2–3. doi: 10.4269/ajtmh.1992.47.512 1443350

[38] Limaye AP, Abrams JS, Silver JE, Awadzi K, Francis HF, Ottesen EA, et al. Interleukin-5 and the posttreatment eosinophilia in patients with onchocerciasis. J Clin Invest. 1991;88(4):1418–21. doi: 10.1172/JCI115449 1918387 PMC295614

[39] Batsa Debrah L, Klarmann-Schulz U, Osei-Mensah J, Dubben B, Fischer K, Mubarik Y, et al. Comparison of Repeated Doses of Ivermectin Versus Ivermectin Plus Albendazole for the Treatment of Onchocerciasis: A Randomized, Open-label, Clinical Trial. Clin Infect Dis. 2020;71(4):933–43. doi: 10.1093/cid/ciz889 31536624 PMC7428389

[40] Luna LG. Manual of histologic staining methods of the Armed Forces Institute of Pathology. 1968.

[41] Bazzocchi C, Ceciliani F, McCall JW, Ricci I, Genchi C, Bandi C. Antigenic role of the endosymbionts of filarial nematodes: IgG response against the Wolbachia surface protein in cats infected with Dirofilaria immitis. Proc Biol Sci. 2000;267(1461):2511–6. doi: 10.1098/rspb.2000.1313 11197127 PMC1690852

[42] Jolodar A, Fischer P, Büttner DW, Miller DJ, Schmetz C, Brattig NW. Onchocerca volvulus: expression and immunolocalization of a nematode cathepsin D-like lysosomal aspartic protease. Exp Parasitol. 2004;107(3–4):145–56. doi: 10.1016/j.exppara.2004.06.006 15363940

[43] Gardon J, Boussinesq M, Kamgno J, Gardon-Wendel N, Demanga-Ngangue, Duke BOL. Effects of standard and high doses of ivermectin on adult worms of Onchocerca volvulus: A randomised controlled trial. Lancet. 2002;360:203–10. doi: 10.1016/S0140-6736(02)09456-4 12133654

[44] Duke BOL, Marty AM, Peett DL, Gardo J, Pion SDS, Kamgno J, et al. Neoplastic change in Onchocerca volvulus and its relation to ivermectin treatment. Parasitology. 2002;125(Pt 5):431–44. doi: 10.1017/s0031182002002305 12458827

[45] Specht S, Brattig N, Büttner M, Büttner DW. Criteria for the differentiation between young and old Onchocerca volvulus filariae. Parasitol Res. 2009;105(6):1531–8. doi: 10.1007/s00436-009-1588-5 19784672 PMC2764059

[46] Büttner DW, Albiez EJ, von Essen J, Erichsen J. Histological examination of adult Onchocerca volvulus and comparison with the collagenase technique. Trop Med Parasitol. 1988;39 Suppl 4:390–417. 2852393

[47] Hoerauf A, Mand S, Volkmann L, Büttner M, Marfo-Debrekyei Y, Taylor M, et al. Doxycycline in the treatment of human onchocerciasis: Kinetics of Wolbachia endobacteria reduction and of inhibition of embryogenesis in female Onchocerca worms. Microbes Infect. 2003;5(4):261–73. doi: 10.1016/s1286-4579(03)00026-1 12706439

[48] QIAamp DNA Mini and Blood Mini Handbook. Qiagen. 2023.

[49] Qiagen. QIAamp DNA Micro Handbook. 2014.

[50] Schlabe S, Korir P, Lämmer C, Landmann F, Dubben B, Koschel M, et al. A qPCR to quantify Wolbachia from few Onchocerca volvulus microfilariae as a surrogate for adult worm histology in clinical trials of antiwolbachial drugs. Parasitol Res. 2022;121(4):1199–206. doi: 10.1007/s00436-021-07411-5 35006317 PMC8986682

[51] Gilbert J, Nfon CK, Makepeace BL, Njongmeta LM, Hastings IM, Pfarr KM, et al. Antibiotic chemotherapy of onchocerciasis: in a bovine model, killing of adult parasites requires a sustained depletion of endosymbiotic bacteria (Wolbachia species). J Infect Dis. 2005;192(8):1483–93. doi: 10.1086/462426 16170768

[52] Osei-Atweneboana MY, Awadzi K, Attah SK, Boakye DA, Gyapong JO, Prichard RK. Phenotypic evidence of emerging ivermectin resistance in Onchocerca volvulus. PLoS Negl Trop Dis. 2011;5(3):e998. doi: 10.1371/journal.pntd.0000998 21468315 PMC3066159

[53] Nana-Djeunga HC, Bourguinat C, Pion SD, Bopda J, Kengne-Ouafo JA, Njiokou F, et al. Reproductive status of Onchocerca volvulus after ivermectin treatment in an ivermectin-naïve and a frequently treated population from Cameroon. PLoS Negl Trop Dis. 2014;8(4):e2824. doi: 10.1371/journal.pntd.0002824 24762816 PMC3998936

[54] Kutu ED, Mensah DA, Opoku VS, Boateng J, Opoku J, Osei-Mensah J, et al. High-Dose Rifampicin Plus Albendazole Rapidly Clears Lymphatic Filariasis Circulating Filarial Antigen in a Randomised Clinical Trial: A Promising Step Toward Short-Course Macrofilaricidal Therapy. Pathogens. 2026;15(2):174. doi: 10.3390/pathogens15020174 41754426 PMC12943464

[55] Sekaggya-Wiltshire C, Dooley KE. Pharmacokinetic and pharmacodynamic considerations of rifamycin antibiotics for the treatment of tuberculosis. Expert Opin Drug Metab Toxicol. 2019;15(8):615–8. doi: 10.1080/17425255.2019.1648432 31339806

[56] Horton J. Albendazole: a review of anthelmintic efficacy and safety in humans. Parasitology. 2000;121 Suppl:S113-32. doi: 10.1017/s0031182000007290 11386684

[57] Ochoa D, Saiz-Rodríguez M, González-Rojano E, Román M, Sánchez-Rojas S, Wojnicz A, et al. High-Fat Breakfast Increases Bioavailability of Albendazole Compared to Low-Fat Breakfast: Single-Dose Study in Healthy Subjects. Front Pharmacol. 2021;12:664465. doi: 10.3389/fphar.2021.664465 33935787 PMC8082448

[58] Lange H, Eggers R, Bircher J. Increased systemic availability of albendazole when taken with a fatty meal. 1988;:315–7. 10.1007/BF00540964 3396623

[59] Polasa K, Krishnaswamy K. Effect of food on bioavailability of rifampicin. 1983;:433–7. 10.1002/j.1552-4604.1983.tb01787.x 6643696

[60] Ceballos L, Nieves E, Juárez M, Aveldaño R, Travacio M, Martos J, et al. Assessment of Diet-Related Changes on Albendazole Absorption, Systemic Exposure, and Pattern of Urinary Excretion in Treated Human Volunteers. Antimicrob Agents Chemother. 2021;65(9):e0043221. doi: 10.1128/AAC.00432-21 34152813 PMC8370216

[61] WHO. Guideline: Alternative mass drug administration regimens to eliminate lymphatic filariasis. Geneva: World Health Organization. 2017. 29565523

[62] Naidoo A, Chirehwa M, Ramsuran V, McIlleron H, Naidoo K, Yende-Zuma N, et al. Effects of genetic variability on rifampicin and isoniazid pharmacokinetics in South African patients with recurrent tuberculosis. Pharmacogenomics. 2019;20(4):225–40. doi: 10.2217/pgs-2018-0166 30767706 PMC6562923

[63] Olafuyi O, Parekh N, Wright J, Koenig J. Inter-ethnic differences in pharmacokinetics-is there more that unites than divides?. Pharmacol Res Perspect. 2021;9(6):e00890. doi: 10.1002/prp2.890 34725944 PMC8561230

[64] Cross HF, Bronsvoort BM, Wahl G, Renz A, Achu-Kwi D, Trees AJ. The entry of ivermectin and suramin into Onchocerca ochengi nodules. Ann Trop Med Parasitol. 1997;91(4):393–401. doi: 10.1080/00034989761012 9290846

[65] Thomas S. Rickettsiales: Biology, Molecular Biology, Epidemiology, and Vaccine Development. Cham: Springer International Publishing. 2016. doi: 10.1007/978-3-319-46859-4

[66] Risch F, Kazakov A, Specht S, Pfarr K, Fischer PU, Hoerauf A, et al. The long and winding road towards new treatments against lymphatic filariasis and onchocerciasis. Trends Parasitol. 2024;40(9):829–45. doi: 10.1016/j.pt.2024.07.005 39122645

[67] Halliday A, Guimaraes AF, Tyrer HE, Metuge HM, Patrick CNW, Arnaud K-OJ, et al. A murine macrofilaricide pre-clinical screening model for onchocerciasis and lymphatic filariasis. Parasit Vectors. 2014;7:472. doi: 10.1186/s13071-014-0472-z 25338621 PMC4212127

[68] Klarmann-Schulz U, Specht S, Debrah AY, Batsa L, Ayisi-Boateng NK, Osei-Mensah J, et al. Comparison of Doxycycline, Minocycline, Doxycycline plus Albendazole and Albendazole Alone in Their Efficacy against Onchocerciasis in a Randomized, Open-Label, Pilot Trial. PLoS Negl Trop Dis. 2017;11(1):e0005156. doi: 10.1371/journal.pntd.0005156 28056021 PMC5215804

[69] Turner JD, Mand S, Debrah AY, Muehlfeld J, Pfarr K, McGarry HF, et al. A randomized, double-blind clinical trial of a 3-week course of doxycycline plus albendazole and ivermectin for the treatment of Wuchereria bancrofti infection. Clin Infect Dis. 2006;42(8):1081–9. doi: 10.1086/501351 16575724

[70] Gunderson EL, Vogel I, Chappell L, Bulman CA, Lim KC, Luo M, et al. The endosymbiont Wolbachia rebounds following antibiotic treatment. McGraw EA, editor. PLoS Pathog. 2020;16: e1008623. doi: 10.1371/journal.ppat.1008623 PMC7371230 32639986

[71] Chappell L, Peguero R, Conner WR, Fowler S, Cooper B, Pfarr K, et al. Fexinidazole and Corallopyronin A target Wolbachia-infected sheath cells present in filarial nematodes. 2025;21(9):e1012929.doi: 10.1371/journal.ppat.1012929 40920875 PMC12443271

[72] Specht S, Pfarr K, Arriens S, Hu M, Klarmann-Schulz U, Koschel M. Combinations of registered drugs reduce treatment times required to deplete Wolbachia in the Litomosoides sigmodontis mouse model. PLoS Negl Trop Dis. 2018;12(1):e0006116. 10.1371/journal.pntd.0006116 29300732 PMC5771630

[73] Hegde S, Marriott AE, Pionnier N, Steven A, Bulman C, Gunderson E, et al. Combinations of the azaquinazoline anti-Wolbachia agent, AWZ1066S, with benzimidazole anthelmintics synergise to mediate sub-seven-day sterilising and curative efficacies in experimental models of filariasis. Front Microbiol. 2024;15:1346068. doi: 10.3389/fmicb.2024.1346068 38362501 PMC10867176

[74] Malik K, Dua A. Albendazole. StatPearls Publishing. 2025. 31971723

[75] Osei-Atweneboana MY, Eng JK, Boakye DA, Gyapong JO, Prichard RK. Prevalence and intensity of Onchocerca volvulus infection and efficacy of ivermectin in endemic communities in Ghana: a two-phase epidemiological study. Lancet. 2007;369(9578):2021–9. doi: 10.1016/S0140-6736(07)60942-8 17574093

[76] WHO/APOC. The World Health Organization year 2010 progress report, 1 September 2009–31 August 2010. Geneva: World Health Organization. 2010.

[77] Boussinesq M, Fobi G, Kuesel AC. Alternative treatment strategies to accelerate the elimination of onchocerciasis. Int Health. 2018;10(suppl_1):i40–8. doi: 10.1093/inthealth/ihx054 29471342 PMC5881258

[78] Venkatesan P. Albendazole. J Antimicrob Chemother. 1998;41:145–7. doi: 10.1093/jac/41.2.145 9533454

[79] Achukwi MD, Harnett W, Renz A. Onchocerca ochengi transmission dynamics and the correlation of O. ochengi microfilaria density in cattle with the transmission potential. Vet Res. 2000;31(6):611–21. doi: 10.1051/vetres:2000144 11129804

[80] Cooper PJ, Mancero T, Espinel M, Sandoval C, Lovato R, Guderian RH, et al. Early human infection with Onchocerca volvulus is associated with an enhanced parasite-specific cellular immune response. J Infect Dis. 2001;183(11):1662–8. doi: 10.1086/320709 11343216

[81] Kamga G-R, Dissak-Delon FN, Nana-Djeunga HC, Biholong BD, Ghogomu SM, Souopgui J, et al. Important progress towards elimination of onchocerciasis in the West Region of Cameroon. Parasit Vectors. 2017;10(1):373. doi: 10.1186/s13071-017-2301-7 28774318 PMC5543544

[82] Pion SDS, Nana-Djeunga HC, Kamgno J, Tendongfor N, Wanji S, Njiokou F, et al. Dynamics of Onchocerca volvulus microfilarial densities after ivermectin treatment in an ivermectin-naïve and a multiply treated population from Cameroon. PLoS Negl Trop Dis. 2013;7(2):e2084. doi: 10.1371/journal.pntd.0002084 23469307 PMC3585010

[83] Taylor MJ, Makunde WH, McGarry HF, Turner JD, Mand S, Hoerauf A. Macrofilaricidal activity after doxycycline treatment of Wuchereria bancrofti: a double-blind, randomised placebo-controlled trial. Lancet. 2005;365(9477):2116–21. doi: 10.1016/S0140-6736(05)66591-9 15964448

[84] Debrah AY, Mand S, Marfo-Debrekyei Y, Batsa L, Pfarr K, Buttner M, et al. Macrofilaricidal effect of 4 weeks of treatment with doxycycline on Wuchereria bancrofti. Trop Med Int Health. 2007;12(12):1433–41. doi: 10.1111/j.1365-3156.2007.01949.x 18076549

[85] Supali T, Djuardi Y, Pfarr KM, Wibowo H, Taylor MJ, Hoerauf A. Doxycycline treatment of Brugia malayi – infected persons reduces microfilaremia and adverse reactions after diethylcarbamazine and albendazole treatment. 2008;46(9):1385–93.doi: 10.1086/586753 18419441

