# Supplementary material for: Assessing the efficacy of high-dose rifampicin plus albendazole against onchocerciasis
Source: PLoS Negl Trop Dis. 2026 Aug 12;20(8):e0014594. doi: 10.1371/journal.pntd.0014594 (PMC13492750; doi:10.1371/journal.pntd.0014594)
Supplement: Supplementary file 1 [file pntd.0014594.s007.docx]

**S1 Table: Primers, Probes and Standards (gBlocks®) sequences used for the real-time qPCR**

| **Type** | **5’ – 3’ Sequences** | **Melting Temperature (Tm ^o^C)** |
| --- | --- | --- |
| wOvFtsZ FW | AGG AAT GGG TGG TGG TAC TG | 60.5 |
| wOvFtsZ RV | CTT TAA CCG CAG CTC TTG CT GTG | 58.4 |
| OvActin FW | GTG CTA CGT TGC TTT GGA CT | 58.4 |
| OvActin RV | GTA ATC ACT TGG CCA TCA GG CGA | 58.4 |
| wOvFtsZ TQP | 6-FAM CCT TGC CGC TTT CGC AAT CAC DDQ1 | 61.0 |
| OvActin TQP | HEX AAC AGG AAA TGG CAA CTG CTGC BHQ-1 | 58.0 |
| wOvFtsZ | CAT ATA AAA GAT AGT CAT ATG CTC TTT ATT **ACAGCAGG AAT GGG TGG TGG TAC TGG TAC AGG TGC AGC ACC AGT GAT TG C GAA AGC GGC AAG GGA AGC AAGAGC TGC GGT TAA AG**AT A AAA TGT TAA AAG AGA AAA AGA TAT TGA CTGTT***** | - |
| OvActin | TTG TTC GTG ACA TCA AAG AAA AGC **TGTG CTA CGT TGC TTT GGA CTT CGA ACA GGA AAT GGC AACTGC TGC ATC GTC ATC GTC TCT CGAAA AAT CTT ATG AAT TGC CTG ATG GCC AAGT GA TTAC** CGT AGG CAA CGA ACG ATT TCG ATG CC***** | - |

**The target sequences are bolded and underlined. The additional sequences are flanking regions from the genomic sequence to achieve the required minimum length of 150 base pairs for the gBlock®. TQP = Taqman Probe, FAM = fluorescein CE-phosphramidite, HEX = hexachlorofluorescein, DDQ1= deep dark quencher 1, BHQ-1 = Black Hole Quencher.*
