# Supplementary material for: Assessing the efficacy of high-dose rifampicin plus albendazole against onchocerciasis
Source: PLoS Negl Trop Dis. 2026 Aug 12;20(8):e0014594. doi: 10.1371/journal.pntd.0014594 (PMC13492750; doi:10.1371/journal.pntd.0014594)
Supplement: Supplementary file 2 [file pntd.0014594.s008.docx]

**S2 Table: Participant compliance with treatment regimens**

| **Treatment group** | **Participants enrolled** | **Completed treatment per protocol** | **Missed dose(s)^a^** | | | | **Failed to complete treatment** |
| --- | --- | --- | --- | --- | --- | --- | --- |
|  |  |  | **one** | **two** | | **three** |  |
| RIF+ALB –  14 Days (TA1) | 30 | 29 | 1 | - | - | | 1**^b^** |
| RIF+ALB –  7 Days (TA2) | 30 | 30 | 1 | 1 | - | | - |
| ALB alone – 14 Days (TA3) | 30 | 29 | 3 | - | 1 | | 1**^c^** |
| No Treatment – Control (TA4) | 30 | 30 | - | - | - | | - |
| Total | 120 | 118 | 5 | 1 | 1 | | 2 |

*TA=Treatment arm; RIF=Rifampicin; ALB=Albendazole*

*^a^Missed consecutive doses resulting from participant absence at scheduled treatment visits*

*^b^High AST and low eGFR*

*^c^High AST and ALT*
