# Supplementary material for: Assessing the efficacy of high-dose rifampicin plus albendazole against onchocerciasis
Source: PLoS Negl Trop Dis. 2026 Aug 12;20(8):e0014594. doi: 10.1371/journal.pntd.0014594 (PMC13492750; doi:10.1371/journal.pntd.0014594)
Supplement: Supplementary file 3 [file pntd.0014594.s009.docx]

**S3 Table: Adverse event assessment**

|  | | **RIF+ALB –**  **14 Days (TA1)** | **RIF+ALB –**  **7 Days (TA2)** | **ALB alone – 14 Days (TA3)** | **No Treatment/Control (TA4)** | **p-value** |
| --- | --- | --- | --- | --- | --- | --- |
| Participant, N | | 30 | 30 | 30 | 30 |  |
| No. of patients with AEs, N (%) | | 15 (50.0) | 18 (60.0) | 4 (13.3) | 1 (3.3) | <0.001^a^ |
| No. of AEs in total, N (%) | | 21/50 (42.0) | 22/50 (44.0) | 6/50 (12.0) | 1/50 (2.0) |  |
| AEs per affected patients | Mean ± SD | 1.40 ± 1.25 | 1.22 ± 0.71 | 1.50 ± 0.87 | 1 ± 0.00 | 0.874^b^ |
|  | Min-Max | 1-6 | 1-4 | 1-3 | 1-1 |  |
|  | Median | 1 | 1 | 1 | 1 |  |
| AEs per total patients | Mean ± SD | 0.70 ± 1.13 | 0.73 ± 0.81 | 0.20 ± 0.60 | 0.03 ± 0.18 | <0.001^b^ |
|  | Min-Max | 0-6 | 0-4 | 0-3 | 0-1 |  |
|  | Median | 0.5 | 1 | 0 | 0 |  |

*N=Number; TA=Treatment arm; RIF=Rifampicin; ALB=Albendazole; AE=Adverse event; SD=Standard deviation*

*^a^Fisher`s exact test*

*^b^Kruskal-Wallis test*

*^b^Multiple comparisons using Dunn’s post-hoc test after the Kruskal-Wallis’s test identified significant differences between the following pairs of groups: TA1 and TA3, p=0.009; TA1 and TA4, p<0.001.TA2 and TA3, p=0.001; TA2 and TA4, p<0.001.*

*Statistics calculated using Stata version 16*
