# Supplementary material for: Assessing the efficacy of high-dose rifampicin plus albendazole against onchocerciasis
Source: PLoS Negl Trop Dis. 2026 Aug 12;20(8):e0014594. doi: 10.1371/journal.pntd.0014594 (PMC13492750; doi:10.1371/journal.pntd.0014594)
Supplement: Supplementary file 4 [file pntd.0014594.s010.docx]

**S4 Table: Adverse event assessment, detailed**

| **Adverse Events** | | | **RIF+ALB – 14 Days (TA1),**  **N (%)** | **RIF+ALB – 7 Days (TA2),**  **N (%)** | **ALB alone – 14 Days (TA3),**  **N (%)** | **No Treatment/Control (TA4), N (%)** | **p-value** |
| --- | --- | --- | --- | --- | --- | --- | --- |
| Reddish coloured urine (due to rifampicin colour— reddish) | No. of AEs | | 13 (61.9) | 16 (72.7) | 0 | 0 | <0.001 |
|  | Grade of AE^*^ | Mild (Grade1) | 7 (53.9) | 10 (62.5) |  |  |  |
|  |  | Moderate (Grade 2) | 6 (46.1) | 6 (35.5) |  |  |  |
|  | Related to treatment^¥^ | Definite | 13 (100) | 16 (100) |  |  |  |
| Headache | No. of AEs | | 1 (4.8) | 2 (9.1) | 1 (16.7) | 0 | 0.901 |
|  | Grade of AE^*^ | Mild (Grade1) | 1 (100) | 2 (100) | 1 (100) |  |  |
|  | Related to treatment^¥^ | Possible | 0 | 0 | 0 |  |  |
|  |  | Not related | 1 (100) | 2 (100) | 1 (100) |  |  |
| Fever | No. of AEs | | 1 (4.8) | 0 | 1 (16.7) | 0 | >0.999 |
|  | Grade of AE^*^ | Mild (Grade1) | 1 (100) |  | 1 (100) |  |  |
|  | Related to treatment^¥^ | Possible | 1 (100) |  | 0 |  |  |
|  |  | Not related | 0 |  | 1 (100) |  |  |
| High ALT | No. of AEs | | 2 (9.5) | 0 | 1 (16.7) | 0 | 0.615 |
|  | Grade of AE^*^ | Moderate (Grade 2) | 2 (100) |  | 1 (100) |  |  |
|  | Related to treatment^¥^ | Possible | 2 (100) |  | 1 (100) |  |  |
| High AST | No. of AEs | | 0 | 0 | 1 (16.7) | 1 (100) | >0.999 |
|  | Grade of AE^*^ | Moderate (Grade 2) |  |  | 1 (100) | 1 (100) |  |
|  | Related to treatmen^¥^ | Possible |  |  | 1 (100) | 0 |  |
|  |  | Not related |  |  | 0 | 1 (100) |  |
| High GGT | No. of AEs | | 1 (4.8) | 0 | 1 (16.7) | 0 | >0.999 |
|  | Grade of AE^*^ | Moderate (Grade 2) | 1 (100) |  | 1 (100) |  |  |
|  | Related to treatment^¥^ | Possible | 1 (100) |  | 1 (100) |  |  |
| Others | No. of AEs | | 3 (14.3)^a^ | 4 (18.2)^b^ | 1 (16.7)^c^ | 0 | 0.753 |
|  | Grade of AE^*^ | Mild (Grade1) | 2 (66.7) | 3 (75.0) | 1 (100) |  |  |
|  |  | Moderate (Grade 2) | 1 (33.3) | 1 (25.0) | 0 |  |  |
|  | Related to treatment^¥^ | Possible | 3(100) | 2 (50.0) | 0 |  |  |
|  |  | Not related | 0 | 2 (50.0) | 1(100) |  |  |
| **Total** | | | 21 (100) | 22 (100) | 6 (100) | 1 (100) |  |

*N=Number; TA=Treatment arm; RIF=Rifampicin; ALB=Albendazole; AE=Adverse event*

*Fisher`s exact test calculated with Stata version 16 (Stata Corporation LLC, College Station, USA)*

*^*^Grading was always “Mild (Grade 1)”, “Moderate (Grade 2)”, “Severe (Grade 3)”. To shorten this table, only the grades which occurred are shown.*

*^¥^Relation to treatment could always be assessed as “definite”, “probable”, “possible”, “remote” and “not related”. To shorten this table only the relations which occurred are shown.*

*^a^ Diarrhoea, Low eGFR (<60* mL/min/1.73m²*), Runny nose*

*^b^ Nausea, Low Hb (<8 g/dL), Low WBC (<3×10^6), Ulceration of the* *hand and face due to motor accident*

*^c^ Stomach ache*
