# Supplementary material for: Assessing the efficacy of high-dose rifampicin plus albendazole against onchocerciasis
Source: PLoS Negl Trop Dis. 2026 Aug 12;20(8):e0014594. doi: 10.1371/journal.pntd.0014594 (PMC13492750; doi:10.1371/journal.pntd.0014594)
Supplement: Supplementary file 5 [file pntd.0014594.s011.docx]

**S5 Table: Effect of treatment on adult worm—PP analysis**

| **Treatment group** | **No. of Patients/Nodules^a^** | **Total no. of worms** | **No. of female worms (%)** | | | **No. of male worms (%)** | | |
| --- | --- | --- | --- | --- | --- | --- | --- | --- |
|  |  |  | *p = 0.184***^c^** | | | *p = 0.872***^d^** | | |
|  |  |  | **Live** | **Dead (%, CI)^b,c^** | **Total** | **Live** | **Dead (%, CI) ^b,d^** | **Total** |
| RIF+ALB –  14 Days (TA1) | 17/38 | 123 | 69 (75.0) | 23  (25.0 [16.6;35.1]) | 92 | 28  (90.3) | 3  (9.7 [2.0;25.8]) | 31 |
| RIF+ALB –  7 Days (TA2) | 21/47 | 155 | 83 (69.8) | 36  (30.3 [22.2;39.3]) | 119 | 35  (97.2) | 1  (2.8 [0.1;14.5]) | 36 |
| ALB alone – 14 Days (TA3) | 20/40 | 120 | 61 (67.0) | 30  (33.0 [23.5;43.6]) | 91 | 27  (93.1) | 2  (6.9 [0.8;22.8]) | 29 |
| No Treatment – Control (TA4) | 21/43 | 136 | 67 (63.8) | 38  (36.2 [27.0; 46.1]) | 105 | 30  (96.8) | 1  (3.2 [0.1;16.7]) | 31 |
| Total | 79/168* | 534 | 280 (68.8) | 127  (31.2 [26.7;36.0]) | 407 | 120  (94.5) | 7  (5.5 [2.2;11.0]) | 127 |

*TA=Treatment arm; PP=per-protocol; RIF=Rifampicin; ALB=Albendazole; CI=Confidence interval*

*^a^Only evaluable patients/nodules are included: Out of the 191 nodules from 89 patients (PP), 23 nodules from 10 patients were not evaluable (non-onchocercal origin, e.g., foreign body granulomas, lipomas or lymph nodes: TA1 = 8, TA2 = 5, TA3 = 9, TA4 = 1)*

*^b^95% confidence intervals for proportions were calculated using the recommended method by Altman et al., (2000)*

*^c^Proc Genmod, SAS^®^ version 9.4: Comparison of all groups (dead female worms vs. live female worms)*

*^d^Proc Genmod, SAS^®^ version 9.4: Comparison of all groups (dead male worms vs. live male worms)*
