# Supplementary material for: Assessing the efficacy of high-dose rifampicin plus albendazole against onchocerciasis
Source: PLoS Negl Trop Dis. 2026 Aug 12;20(8):e0014594. doi: 10.1371/journal.pntd.0014594 (PMC13492750; doi:10.1371/journal.pntd.0014594)
Supplement: Supplementary file 6 [file pntd.0014594.s012.docx]

**S6 Table: Effect of treatment on adult worms—ITT analysis**

| **Treatment group** | **Number of Patients/Nodules^a^** | **Total number of worms** | **No. of female worms** | | | **No. of male worms** | | |
| --- | --- | --- | --- | --- | --- | --- | --- | --- |
|  |  |  | *p = 0.2192^c^* | | | *p = 0.8384^d^* | | |
|  |  |  | **Live (%)** | **Dead (%, CI)^b,c^** | **Total** | **Live (%)** | **Dead (%, CI) ^b,d^** | **Total** |
| RIF+ALB –  14 Days (TA1) | 20/45 | 141 | 77 (74.0) | 27  (26.0 [17.9; 35.5]) | 104 | 34  (91.9) | 3  (8.1 [1.7;21.9]) | 37 |
| RIF+ALB –  7 Days (TA2) | 22/52 | 166 | 89  (70.6) | 37  (29.4 [21.6;38.1]) | 126 | 38 (95.0) | 2  (5.0 [0.6;16.9]) | 40 |
| ALB alone – 14 Days (TA3) | 25/48 | 140 | 71 (66.4) | 36  (33.6 [24.8;43.4]) | 107 | 30  (90.9) | 3  (9.1 [1.9;24.3]) | 33 |
| No Treatment/Control (TA4) | 25/51 | 149 | 75 (65.8) | 39  (34.2 [25.6;43.7]) | 114 | 34  (97.1) | 1  (2.9 [0.1;14.9]) | 35 |
| Total | 92/196 | 596 | 312 (69.2) | 139  (30.8 [26.7;35.2]) | 451 | 136  (93.8) | 9  (6.2 [2.9;11.5]) | 145 |

*TA=Treatment arm; ITT=Intention-to-treat; RIF=Rifampicin; ALB=Albendazole; CI=Confidence interval*

*^a^Only evaluable patients/nodules are included: Out of the 223 nodules from 105 patients (ITT), 27 nodules from 13 patients were not evaluable* *(non-onchocercal origin e.g., foreign body granulomas, lipomas or lymph nodes: TA1 = 7, TA2 = 9, TA3 = 10, TA4 = 1)*

*^b^95% confidence intervals for proportions were calculated using the recommended method by Altman et al. (2000)*

*^c^Proc Genmod, SAS^®^ version 9.4: Comparison of all groups (dead female worms vs. live female worms)*

*^d^Proc Genmod, SAS^®^ version 9.4: Comparison of all groups (dead male worms vs. live male worm*
