# Supplementary material for: Assessing the efficacy of high-dose rifampicin plus albendazole against onchocerciasis
Source: PLoS Negl Trop Dis. 2026 Aug 12;20(8):e0014594. doi: 10.1371/journal.pntd.0014594 (PMC13492750; doi:10.1371/journal.pntd.0014594)
Supplement: Supplementary file 7 [file pntd.0014594.s013.docx]

**S7 Table: Effect of treatment on embryogenesis—PP analysis**

| **Treatment group** | **Number of Patients/**  **Nodules^a^** | **Number of living female worms*** | | | | | | | |
| --- | --- | --- | --- | --- | --- | --- | --- | --- | --- |
|  |  | **Embryogenesis (%)^¥^** | | | | | | | **Sperms in uterus (%)**** |
|  |  | **Judgement not possible** | **Debris only** | **Uterus empty** | **Oocytes only** | **Normal embryogenesis** | **Degenerated embryogenesis** | **Total*** |  |
| RIF+ALB –  14 Days (TA1) | 17/38 | 1 | 0 | 0 (0.0) | 35 (55.6) | 18 (28.6) | 10 (15.9) | 64 | 11 (17.2) |
| RIF+ALB –  7 Days (TA2) | 21/47 | 5 | 1 | 0 (0.0) | 40 (54.8) | 24 (32.9) | 9 (12.3) | 79 | 10 (12.7) |
| ALB alone – 14 Days (TA3) | 20/40 | 7 | 2 | 0 (0.0) | 26 (53.1) | 16 (32.7) | 7 (14.3) | 58 | 9 (15.5) |
| No Treatment/  Control (TA4) | 21/43 | 3 | 1 | 1 (1.7) | 33 (55.9) | 18 (30.5) | 7 (11.9) | 63 | 9 (14.3) |
| Total | 79/168 | 16 | 4 | 1(0.4) | 134 (54.9) | 76 (31.1) | 33 (13.5) | 264 | 39 (14.8) |

*TA=Treatment arm; PP=per-protocol*

*^a^*Only evaluable patients/nodules are included: Out of the 191 nodules from 89 patients (PP), 23 nodules from 10 patients were not evaluable (non-onchocercal origin, e.g., foreign body granulomas, lipomas or lymph nodes: TA1 = 8, TA2 = 5, TA3 = 9, TA4 = 1)*

***280 living female worms examined, 16 neoplastic: ‘Total’ excludes neoplastic.*

*^¥^Percentage computations for embryogenesis excludes judgement not possible and debris only.*

***Calculated based on the total number of living female worms, excluding neoplastic worms.*
