# Supplementary material for: Assessing the efficacy of high-dose rifampicin plus albendazole against onchocerciasis
Source: PLoS Negl Trop Dis. 2026 Aug 12;20(8):e0014594. doi: 10.1371/journal.pntd.0014594 (PMC13492750; doi:10.1371/journal.pntd.0014594)
Supplement: Supplementary file 8 [file pntd.0014594.s014.docx]

**S8 Table: Effect of treatment on embryogenesis: statistics^a,b^—PP analysis**

| **Female** | **RIF+ALB – 7 Days (TA1)** | **ALB alone – 14 Days (TA3)** | **No Treatment/Control (TA4)** |
| --- | --- | --- | --- |
| RIF+ALB –  14 Days (TA1) | OR 0.6860 [0.22;2.18]  *p* = 0.523 | OR 0.8076 [0.22;3.02]  *p* = 0.751 | OR 0.7112 [0.20;2.48]  *p* = 0.593 |
| RIF+ALB –  7 Days (TA2) |  | OR 1.1610 [0.32;4.18]  *p* = 0.819 | OR 1.0354 [0.31;3.50]  *p* = 0.955 |
| ALB alone – 14 Days (TA3) |  |  | OR 0.8898 [0.23;3.48]  *p* = 0.867 |

*TA=Treatment arm; PP=per-protocol*

*^a^**Alternating logistic regression: comparison of normal vs. degenerated embryogenesis only, calculated using SAS^®^ version 9.4*

*^b^Odds ratios (OR) showing the presence of embryogenesis: compares row treatment groups to the column treatment groups.*
