# Supplementary material for: Assessing the efficacy of high-dose rifampicin plus albendazole against onchocerciasis
Source: PLoS Negl Trop Dis. 2026 Aug 12;20(8):e0014594. doi: 10.1371/journal.pntd.0014594 (PMC13492750; doi:10.1371/journal.pntd.0014594)
Supplement: Supplementary file 9 [file pntd.0014594.s015.docx]

| **Treatment group** | **Number of Patients/Nodules^a^** |  | **Number of living female worms*** | | | | | | |
| --- | --- | --- | --- | --- | --- | --- | --- | --- | --- |
|  |  |  | **Embryogenesis (%)^¥^** | | | | | | **Sperms in uterus (%)**** |
|  |  | **Judgement not possible** | **Debris only** | **Uterus empty** | **Oocytes only** | **Normal embryogenesis** | **Degenerated embryogenesis** | **Total*** |  |
| RIF+ALB –  14 Days (TA1) | 20/45 | 2 | 0 | 0 (0.0) | 39 (56.5) | 20 (29.0) | 10 (14.5) | 71 | 12 (16.9) |
| RIF+ALB –  7 Days (TA2) | 22/52 | 5 | 1 | 0 (0.0) | 44 (55.7) | 26 (32.9) | 9 (11.4) | 85 | 11 (12.9) |
| ALB alone – 14 Days (TA3) | 25/48 | 7 | 2 | 0 (0.0) | 30 (51.7) | 17 (29.3) | 11 (19.0) | 67 | 12 (17.9) |
| No Treatment/Control (TA4) | 25/51 | 3 | 1 | 1 (1.5) | 37 (56.1) | 19 (28.8) | 9 (13.6) | 70 | 11 (15.7) |
| Total | 92/196 | 17 | 4 | 1 (0.4) | 150 (55.2) | 82 (30.1) | 39 (14.3) | 293 | 46 (15.7) |

**S9 Table: Effect of treatment on embryogenesis—ITT analysis**

*TA=Treatment arm; ITT=Intention-to-treat*

*^a^ Only evaluable patients/nodules are included: Out of the 223 nodules from 105 patients (ITT), 27 nodules from 13 patients were not evaluable (non-onchocercal origin, e,g. foreign body granulomas, lipomas or lymph nodes: TA1 = 7, TA2 = 9, TA3 = 10, TA4 = 1)*

**312 living female worms examined, 19 neoplastic:‘Total’ excludes neoplastic.*

*^¥^Percentage computations for embryogenesis excludes judgement not possible and debris only.*

***Calculated based on the total number of living female worms, excluding neoplastic worms.*
