# Supplementary material for: Assessing the efficacy of high-dose rifampicin plus albendazole against onchocerciasis
Source: PLoS Negl Trop Dis. 2026 Aug 12;20(8):e0014594. doi: 10.1371/journal.pntd.0014594 (PMC13492750; doi:10.1371/journal.pntd.0014594)
Supplement: Supplementary file 10 [file pntd.0014594.s016.docx]

| **Female** | **RIF+ALB – 7 Days (TA2)** | **ALB alone – 14 Days (TA3)** | **No Treatment/Control (TA4)** |
| --- | --- | --- | --- |
| RIF+ALB –  14 Days (TA1) | OR 0.7020 [0.22;2.19]  *p* = 0.5426 | OR 13240 [0.37;4.72]  *p* = 0.6652 | OR 0.9938 [0.28;3.56]  *p* = 0.9924 |
| RIF+ALB –  7 Days (TA2) |  | OR 18355 [0.54;6.18]  *p* = 0.3272 | OR 13882 [0.41;4.71]  *p* = 0.5992 |
| ALB alone – 14 Days (TA3) |  |  | OR 0.7533 [0.20;2.83]  *P* = 0.6746 |

**S10 Table: Effect of treatment on embryogenesis: statistics^a,b^–ITT analysis**

*TA=Treatment arm; ITT=Intention-to-treat*

*^a^Alternating logistic regression: comparison of normal vs. degenerated embryogenesis only, calculated using SAS^®^ version 9.4*

*^b^Odds ratios (OR) for the presence of embryogenesis:* *compares row treatment groups to the column treatment groups*
