# Supplementary material for: Assessing the efficacy of high-dose rifampicin plus albendazole against onchocerciasis
Source: PLoS Negl Trop Dis. 2026 Aug 12;20(8):e0014594. doi: 10.1371/journal.pntd.0014594 (PMC13492750; doi:10.1371/journal.pntd.0014594)
Supplement: Supplementary file 11 [file pntd.0014594.s017.docx]

**S11 Table: Effect of treatment on intranodular microfilariae—PP analysis**

| **Treatment group** | **Number of Patients/Nodules^a^** | **Number of evaluable nodules with live female worms or intact intranodular microfilariae (MFs)^b^** | |
| --- | --- | --- | --- |
|  |  | **Total** | **with intact MF (% [CI])^c,d^** |
|  |  |  | *p = 0.571^d^* |
| RIF+ALB –  14 Days (TA1) | 17/38 | 34 | 9 (26.5 [12.9;44.4]) |
| RIF+ALB –  7 Days (TA2) | 21/47 | 41 | 14 (34.1 [20.1;50.6]) |
| ALB alone – 14 Days (TA3) | 20/40 | 34 | 8 (23.5 [10.7;41.2]) |
| No Treatment/Control (TA4) | 21/42 | 37 | 10 (27.0[13.8;44.1]) |
| Total | 79/167*^¥^ | 146 | 41 (28.1 [21.0; 36.1]) |

*TA=Treatment arm; PP=per-protocol; RIF=Rifampicin; ALB=Albendazole; CI=Confidence interval; MF=Microfilaria*

*^a*^Only evaluable patients/nodules are included: Out of the 191 nodules from 89 patients (PP), 23 nodules from 10 patients were not evaluable (non-onchocercal origin, e,g., foreign body granulomas, lipomas or lymph nodes: TA1 = 5, TA2 = 8,TA3 = 9, TA4 = 1)*

*^¥^1 missing in TA4 for intact MFs*

*^b^Only nodules with living female worms or intact MFs are included*

*^c^95% confidence intervals (CI) for proportions were calculated using the recommended method by Altman et al., (2000)*

*^d^Proc Genmod, SAS^®^ version 9.4: Comparison of all groups (nodules with intact MF vs. all other evaluable nodules)*
