# Supplementary material for: Assessing the efficacy of high-dose rifampicin plus albendazole against onchocerciasis
Source: PLoS Negl Trop Dis. 2026 Aug 12;20(8):e0014594. doi: 10.1371/journal.pntd.0014594 (PMC13492750; doi:10.1371/journal.pntd.0014594)
Supplement: Supplementary file 12 [file pntd.0014594.s018.docx]

**S12** T**able: Effect of treatment on intranodular microfilariae (MF)—ITT analysis**

| **Treatment group** | **Number of Patients/Nodules^a^** | **Number of evaluable nodules with live female worms or intact intranodular MFs^b^** | |
| --- | --- | --- | --- |
|  |  | **Total** | **with intact MF (%, CI)^c,d^** |
|  |  |  | *p = 0.6257^d^* |
| RIF+ALB –  14 Days (TA1) | 20/45 | 40 | 9  (22.5 [10.8;38.5]) |
| RIF+ALB –  7 Days (TA2) | 22/52 | 45 | 15  (33.3[20.0;49.0]) |
| ALB alone – 14 Days (TA3) | 25/48 | 40 | 8  (20.0 [9.1;35.6]) |
| No Treatment/Control (TA4) | 25/50 | 45 | 11 (24.4 [12.9;39.5]) |
|  | 92/195^*^ | 170 | 43 (25.3 [19.0; 32.5]) |

*TA=Treatment arm; ITT=Intention-to-treat; RIF=Rifampicin; ALB=Albendazole; CI=Confidence interval; MF=Microfilaria*

*^a^ Only evaluable patients/nodules are included: Out of the 223 nodules from 105 patients, 27 nodules from 13 patients were not evaluable (non-onchocercal origin e.g., foreign body granulomas, lipomas or lymph nodes: TA2 = 7, TA1 = 9, TA3 = 10, TA4 = 1)*

*^b^ Only nodules with living female worms or intact MFs are included*

*^c^95% confidence intervals (CI) for proportions were calculated using the recommended method by Altman et al. (2000)^*^1 missing in TA4 for intact MFs*

*^d^Proc Genmod, SAS^®^ version 9.4: Comparison of all groups (nodules with intact MF vs. all other evaluable nodules).*
