# Supplementary material for: Assessing the efficacy of high-dose rifampicin plus albendazole against onchocerciasis
Source: PLoS Negl Trop Dis. 2026 Aug 12;20(8):e0014594. doi: 10.1371/journal.pntd.0014594 (PMC13492750; doi:10.1371/journal.pntd.0014594)
Supplement: Supplementary file 13 [file pntd.0014594.s019.docx]

**S13 Table: Effect of treatment on the presence of Wolbachia in female worms: immunohistology—PP analysis**

| **Treatment Group** | **No. of Patients/Nodules^a^** | **No. of living female worms*** | | | | **Alternating logistic regression^b,c**^** | |
| --- | --- | --- | --- | --- | --- | --- | --- |
|  |  | ***Wolbachia* levels (%)** | | | |  |  |
|  |  | **Many** | **Few** | **None** | **Total** | **RIF+ALB – 7 Days (TA2)** | **No Treatment/Control (TA4)** |
| RIF+ALB –14 Days (TA1) | 17/38 | 40 (63.5) | 20 (31,8) | 3 (4.8) | 63 | OR 4.0294 [0.40;40.51]  *p* = 0.237 | OR 2.8374 [0.25;31.84]  *p* = 0.398 |
| RIF+ALB –7 Days (TA2) | 21/47 | 38 (48.1) | 40 (50.6) | 1 (1.3) | 79 |  | OR 0.7081 [0.05;11.00]  *p* = 0.8051 |
| ALB alone – 14 Days (TA3) | 20/40 | 29 (50.0) | 29 (50.0) | 0 (0.0) | 58 |  |  |
| No Treatment/  Control (TA4) | 21/43 | 28 (44.4) | 34 (54.0) | 1 (1.6) | 63 |  |  |
| Total | 79/168 | 135 (51.3) | 123 (46.8) | 5 (1.9) | 263 |  |  |

*TA=Treatment arm; PP=per-protocol; RIF=Rifampicin; ALB=Albendazole*

*^a^Only evaluable patients/nodules are included: Out of the 191 nodules from 89 patients (PP), 23 nodules from 10 patients were not evaluable (non-onchocercal origin, e.g., foreign body granulomas, lipomas or lymph nodes: TA1 = 8, TA2 = 5, TA3 = 9, TA4 = 1)*

**280 living female worms examined, 16 neoplastic, 1 with judgement of Wolbachia not possible due to poor worm quality.*

*^b^Comparison of presence (many and few) vs. absence of Wolbachia*

*^c^Odds ratios (OR) showing the presence of Wolbachia: compares row treatment groups to the column treatment groups.*

***ALB alone – 14 Days (TA3) group was not included in the analysis because it had only female worms with Wolbachia.*

*Alternating logistic regression calculated using SAS^®^ version 9.4*
