# Supplementary material for: Assessing the efficacy of high-dose rifampicin plus albendazole against onchocerciasis
Source: PLoS Negl Trop Dis. 2026 Aug 12;20(8):e0014594. doi: 10.1371/journal.pntd.0014594 (PMC13492750; doi:10.1371/journal.pntd.0014594)
Supplement: Supplementary file 14 [file pntd.0014594.s020.docx]

| **Treatment Group** | **No. of Patients/Nodules^a^** | **No. of living female worms*** | | | | **Alternating logistic regression^b,c**^** | |
| --- | --- | --- | --- | --- | --- | --- | --- |
|  |  | ***Wolbachia* levels (%)** | | | |  |  |
|  |  | **Many** | **Few** | **None** | **Total** | **RIF+ALB –7 Days (TA2)** | **No Treatment/Control (TA4)** |
| RIF+ALB –14 Days (TA1) | 20/45 | 45 (64.3) | 22 (31.4) | 3 (4.3) | 70 | OR 3.8745 [0.38;39.24]  *p* = 0.2516 | OR 2.7953 [0.25;31.19]  *p* = 0.4036 |
| RIF+ALB –7 Days (TA2) | 22/52 | 42 (49.4) | 42 (49.4) | 1 (1.2) | 85 |  | OR 0.7256 [0.05;11.24]  *p* = 0.8186 |
| ALB alone – 14 Days (TA3) | 25/48 | 35 (52.2) | 32 (47.8) | 0 (0.0) | 67 |  |  |
| No Treatment/Control (TA4) | 25/51 | 34 (48.6) | 35 (50.0) | 1 (1.4) | 70 |  |  |
| Total | 92/196* | 156 (53.4) | 131 (44.9) | 5 (1.7) | 292 |  |  |

**S14 Table: Effect of treatment on the presence of Wolbachia in female worms: immunohistology—ITT analysis**

*TA=Treatment arm; ITT=Intention-to-treat*

*^a*^Only evaluable patients/nodules are included: Out of the 223 nodules from 105 patients (ITT), 27 nodules from 13 patients were not evaluable (non-onchocercal origin e.g., foreign body granulomas, lipomas or lymph nodes: TA1 = 7, TA2 = 9, TA3 = 10, TA4 = 1)*

**312 living female worms examined, 19 neoplastic, 1 with judgement of Wolbachia not possible due to poor worm quality.*

*^b^Comparison of presence (many and few) vs. absence of Wolbachia*

*^c^Odds ratios (OR) showing the presence of Wolbachia: compares row treatment groups to the column treatment groups.*

***ALB alone – 14 Days (TA3) group was not included in the analysis because it had no Wolbachia.*

*Alternating logistic regression calculated using SAS^®^ version 9.4*
