# Supplementary material for: Assessing the efficacy of high-dose rifampicin plus albendazole against onchocerciasis
Source: PLoS Negl Trop Dis. 2026 Aug 12;20(8):e0014594. doi: 10.1371/journal.pntd.0014594 (PMC13492750; doi:10.1371/journal.pntd.0014594)
Supplement: Supplementary file 15 [file pntd.0014594.s021.docx]

**S15 Table: Effect of treatment on the presence of Wolbachia in male worms: immunohistology—PP analysis**

| **Treatment Group** | **No. of Patients/Nodules^a^** | **No. of living male worms*** | | | | **Alternating logistic regression^b,c**^** |
| --- | --- | --- | --- | --- | --- | --- |
|  |  | ***Wolbachia* levels (%)** | | | |  |
|  |  | **Many** | **Few** | **None** | **Total** | **RIF+ALB – 7 Days (TA2)** |
| RIF+ALB –14 Days (TA1) | 17/38 | 18 (66.7) | 8 (29.6) | 1 (3.7) | 27 | OR 1.2463 [0.08;20.01]  *p* = 0.877 |
| RIF+ALB –7 Days (TA2) | 21/47 | 15 (46.9) | 16 (50.0) | 1 (3.1) | 32 |  |
| ALB alone – 14 Days (TA3) | 20/40 | 13 (48.2) | 14 (51.9) | 0 (0.0) | 27 |  |
| No Treatment/Control (TA4) | 21/43 | 18 (64.3) | 10 (35.7) | 0 (0.0) | 28 |  |
| Total | 79/168 | 64 (56.1) | 48 (42.1) | 2 (1.8) | 114 |  |

*TA=Treatment arm; PP=per-protocol; RIF=Rifampicin; ALB=Albendazole*

*^a^Only evaluable patients/nodules are included: Out of the 191 nodules from 89 patients (PP), 23 nodules from 10 patients were not evaluable (non-onchocercal origin, e,g., foreign body granulomas, lipomas or lymph nodes: TA1 = 8, TA2 = 5, TA3 = 9, TA4 = 1)*

**120 living male worms examined, 1 was neoplastic (TA), 5 with judgement of Wolbachia not possible due to poor worm quality.*

*^b^Comparison of presence (many and few) vs. absence of Wolbachia*

*^c^Odds ratios (OR) showing the presence of Wolbachia: compares row treatment groups to the column treatment groups.*

***ALB alone – 14 Days (TA3) group and No treatment/Control (TA4) were not included in the analysis because both had no Wolbachia.*

*Alternating logistic regression calculated using SAS^®^ version 9.4*
