# Supplementary material for: Assessing the efficacy of high-dose rifampicin plus albendazole against onchocerciasis
Source: PLoS Negl Trop Dis. 2026 Aug 12;20(8):e0014594. doi: 10.1371/journal.pntd.0014594 (PMC13492750; doi:10.1371/journal.pntd.0014594)
Supplement: Supplementary file 16 [file pntd.0014594.s022.docx]

**S16 Table: Effect of treatment on the presence of Wolbachia in male worms: immunohistology—ITT analysis**

| **Treatment Group** | **No. of Patients/Nodules^a^** | **No. of living male worms*** | | | | **Alternating logistic regression^b,c**^** |
| --- | --- | --- | --- | --- | --- | --- |
|  |  | ***Wolbachia* levels (%)** | | | |  |
|  |  | Many | Few | None | Total | **RIF+ALB –7 Days (TA2)** |
| RIF+ALB –14 Days (TA1) | 20/45 | 21 (63.7) | 11 (33.3) | 1 (3.0) | 33 | OR 1.1037 [0.07;17.79]  *p* = 0.9445 |
| RIF+ALB –7 Days (TA2) | 22/52 | 17 (48.6) | 17 (48.6) | 1 (2.8) | 35 |  |
| ALB alone – 14 Days (TA3) | 25/48 | 14 (48.3) | 15 (51.7) | 0 (0.0) | 29 |  |
| No Treatment/Control (TA4) | 25/51 | 20 (64.5) | 11 (35.5) | 0 (0.0) | 31 |  |
| Total | 92/196* | 72 (56.2) | 54 (42.2) | 2 (1.6) | 128 |  |

*TA=Treatment arm; ITT=Intention-to-treat; RIF=Rifampicin; ALB=Albendazole*

*^a^Only evaluable patients/nodules are included: Out of the 223 nodules from 105 patients (ITT), 27 nodules from 13 patients were not evaluable (non-onchocercal origin e.g., foreign body granulomas, lipomas or lymph nodes; TA1 = 7, TA2 = 9, TA3 = 10, TA4 = 1)*

**136 living male worms examined, 2 neoplastic, 6 with judgement of Wolbachia not possible due to poor worm quality.*

*^b^Comparison of presence (many and few) vs. absence of Wolbachia*

*^c^Odds ratios (OR) showing the presence of Wolbachia: compares row treatment groups to the column treatment groups.*

***ALB alone – 14 Days (TA3) group and No treatment/Control (TA4) were not included in the analysis because both had no Wolbachia*

*Alternating logistic regression calculated using SAS^®^ version 9.4*
