# Supplementary material for: Assessing the efficacy of high-dose rifampicin plus albendazole against onchocerciasis
Source: PLoS Negl Trop Dis. 2026 Aug 12;20(8):e0014594. doi: 10.1371/journal.pntd.0014594 (PMC13492750; doi:10.1371/journal.pntd.0014594)
Supplement: Supplementary file 17 [file pntd.0014594.s023.docx]

**S17 Table: Selection of skin snip samples for qPCR and Wolbachia depletion analysis**—ITT analysis**.**

|  | | **RIF+ALB –**  **14 Days (TA1)** | | **RIF+ALB –**  **7 Days (TA2)** | | **ALB alone – 14 Days (TA3)** | | **No Treatment/Control (TA4)** | | **P-value^a^** | |  |
| --- | --- | --- | --- | --- | --- | --- | --- | --- | --- | --- | --- | --- |
| **Baseline** |  | | | | | | | | | | | |
| Participants | N | | 30 | | 30 | | 30 | | 30 | |  | |
| Skin Snips | Total, N | | 60 | | 60 | | 60 | | 60 | |  | |
| MF Positive | N (%) | | 46 (76.7) | | 52 (86.7) | | 55 (91.7) | | 51 (85.0) | | 0.155 | |
| **4 Months** |  | | | | | | | | | | | |
| Participants | N | | 30 | | 28 | | 29 | | 28 | |  | |
| Skin Snips | Total, N | | 60 | | 56 | | 58 | | 56 | |  | |
| MF Positive | N (%) | | 42 (70.0) | | 42 (75.0) | | 45 (77.6) | | 49 (87.5) | | 0.135 | |
| OvActin Positive^†^ | SS^†^, N (%) | | 41 (97.6) | | 40 (95.2) | | 44 (97.8) | | 49 (100.0) | | 0.417 | |
| *w*OvFtsZ Positive^†^ | SS^†^, N (%) | | 37 (90.2) | | 36 (90.0) | | 38 (86.4) | | 41 (83.7) | | 0.780 | |

*N=Number; TA=Treatment arm; RIF=Rifampicin; ALB=Albendazole; ITT=Intention-to-treat*

*^†^Only skin snips with at least 1 MF count and a positive OvActin signal were used for analysis.*

*^a^Fisher’s exact test calculated using Stata version 16*
