# Supplementary material for: Assessing the efficacy of high-dose rifampicin plus albendazole against onchocerciasis
Source: PLoS Negl Trop Dis. 2026 Aug 12;20(8):e0014594. doi: 10.1371/journal.pntd.0014594 (PMC13492750; doi:10.1371/journal.pntd.0014594)
Supplement: Supplementary file 18 [file pntd.0014594.s024.docx]

**S18 Table: Effect of treatment on the presence of Wolbachia in nodule sections: qPCR^a^—PP analysis**

|  | | **RIF+ALB –**  **14 Days (TA1)** | **RIF+ALB –**  **7 Days (TA1)** | **ALB alone – 14 Days (TA3)** | **No Treatment/Control (TA4)** |
| --- | --- | --- | --- | --- | --- |
| *w*OvFtsZ | N | 25 | 29 | 22 | 30 |
|  | Median | 44445 | 48365 | 45732.50 | 12845 |
|  | 95% CI (Median) | 9747.72;97187.25 | 29629.34;65246,99 | 1642.37;79510.12 | 7430.14;35284.01 |
|  | Percentiles  25^th^; 75th | 8463;115350 | 25050;83610 | 1200;82850 | 4574.50;39247.50 |
|  | Min-Max | 246.75-210600 | 34.84-168300 | 36.94-191900 | 131.90-289250 |
| OvActin | N | 25 | 29 | 22 | 30 |
|  | Median | 11040 | 16750 | 9163.75 | 5610 |
|  | 95% CI (Median) | 4074.08;26903.14 | 5489.17;36879.79 | 5285.07;64586.75 | 2660.52;13969 |
|  | Percentiles  25^th^, 75th | 3938.50;27690 | 3606;47795 | 2735.50;78030 | 2093;19755 |
|  | Min-Max | 447.05-95320 | 62.50;1227000 | 232.1-433800 | 326.40-750300 |
| *w*OvFtsZ/ OvActin | N | 25 | 29 | 22 | 30 |
|  | Median | 2.44 | 2.20 | 1.35 | 1.71 |
|  | 95% CI (Median) | 0.82;10.13 | 1.26;7.89 | 0.71;5.58 | 0.69;4.28 |
|  | Percentiles  25^th^, 75th | 0.67;14.49 | 0.77;8.46 | 0.40;6.01 | 0.51;5.99 |
|  | Min-Max | 0,04-51.99 | 0-145.71 | 0-99.37 | 0.02-41.24 |
| *w*OvFtsZ/ OvActin | %Change^b^ | -42.69 | -28.65 | 21.05 | 0.00 |

*N=Number; CI=Confidence interval; TA=Treatment arm; PP=per-protocol; RIF=Rifampicin; ALB=Albendazole*

*^a^For this analysis, qPCR results from nodules with dead worms only or without an actin signal were excluded*

*^b^Percentage change was calculated by using the No Treatment/Control arm as reference*

*Descriptive statistics (median, minimum, maximum, percentiles, 95% CI, and percentage change) were calculated using Stata version 16*
