# Supplementary material for: Assessing the efficacy of high-dose rifampicin plus albendazole against onchocerciasis
Source: PLoS Negl Trop Dis. 2026 Aug 12;20(8):e0014594. doi: 10.1371/journal.pntd.0014594 (PMC13492750; doi:10.1371/journal.pntd.0014594)
Supplement: Supplementary file 19 [file pntd.0014594.s025.docx]

**S19 Table: Effect of treatment on the presence of Wolbachia in nodule sections: statistics^a,b^—PP analysis**

| **Comparison** | **wOvFtsZ** | **OvActin** | **wOvFtsZ/OvActin** |
| --- | --- | --- | --- |
| TA1 vs TA2 | OR 1.4255 [0.82;2.49]  *p* = 0.2137 | OR 1.1941 [0.67;2.13]  *p* = 0.5493 | OR 1.1417 [0.76; 1.71]  *p* = 0.5182 |
| TA1 vs TA3 | OR 1.4743 [0.86;2.52]  *p* = 0.1558 | OR 0.9185 [0.51;1.64]  *p* = 0.7740 | OR 1.2808 [0.84;1.96]  *p* = 0.2521 |
| TA1 vs TA 4 | OR 1.5233 [1.03;2.26]  *p* = 0.0358 | OR 1.1029 [0.73;1.67]  *p* = 0.6449 | OR 1.2537 [0.86;1.82]  *p* = 0.2356 |
| TA2 vs TA3 | OR 1.0394 [0.53; 2.05]  *p* = 0.9112 | OR 0.7691 [0.38;2.57]  *p* = 0.4699 | OR 1.1212 [0.72;1.74]  *p* = 0.6116 |
| TA2 vs TA4 | OR 1.0778 [0.61;1.90]  *p* = 0.7960 | OR 0.9226 [0.51;1.68]  *p* = 0.7922 | OR 1.0959 [0.74;1.62]  *p* = 0.6477 |
| TA3 vs TA4 | OR 1.0350 [O.59;1.81]  *p* = 0.9036 | OR 1.1935 [0.66;2.15]  *p* = 0.5572 | OR 0.9791 [0.65;1.48]  *p* = 0.9205 |

*TA=Treatment arm; PP=per-protocol; TA1=RIF+ALB- 14 days; TA2=RIF+ALB- 7 days; TA3=ALB alone- 14 days; TA4=No treatment/control*

*^a^Alternating linear regression after log10-transformation (0.1 was added to all values to circumvent zero values)*

*^b^Odds ratios (OR) showing the presence of Wolbachia: pairwise comparisons between treatment groups as specified in the ‘Comparison’ column*

*Alternating linear regression calculated using SAS^®^ version 9.4*
