# Supplementary material for: Assessing the efficacy of high-dose rifampicin plus albendazole against onchocerciasis
Source: PLoS Negl Trop Dis. 2026 Aug 12;20(8):e0014594. doi: 10.1371/journal.pntd.0014594 (PMC13492750; doi:10.1371/journal.pntd.0014594)
Supplement: Supplementary file 20 [file pntd.0014594.s026.docx]

|  |  | **RIF+ALB –**  **14 Days (TA1)** | **RIF+ALB –**  **7 Days (TA2)** | **ALB alone – 14 Days (TA3)** | **No Treatment/Control (TA4)** |
| --- | --- | --- | --- | --- | --- |
| *w*OvFtsZ | N | 31 | 32 | 29 | 38 |
|  | Median | 43155 | 46877.50 | 16975 | 12845 |
|  | 95% CI (Median) | 12554.55;83736 | 28009.87;62634.42 | 1721.93;72433.85 | 7655.39;36549.98 |
|  | Percentiles 25^th;^ 75^th^ | 6152.50;115350 | 23062.50;88845 | 1675.50; 78465 | 4574.50;40290 |
|  | Min-Max | 246.75-404000 | 34.84-554050 | 0.00-191900 | 131.90-1552000 |
| OvActin | N | 31 | 32 | 29 | 38 |
|  | Median | 6510 | 12282 | 7099.5 | 5610 |
|  | 95% CI (Median) | 4777.40;18070.62 | 4062.44;36410.29 | 2407.28;24752.92 | 2711.87;14942.39 |
|  | Percentiles 25^th^, 75^th^ | 3510;27100 | 3027;52127.50 | 1580;54290 | 2093;39665 |
|  | Min-Max | 447.05-95320 | 62.50-1227000 | 219-433800 | 326.40-750300 |
| *w*OvFtsZ/ OvActin | N | 31 | 32 | 29 | 38 |
|  | Median | 4.34 | 2.28 | 1.45 | 1.76 |
|  | 95% CI (Median) | 1.32;9.29 | 1.35;5.33 | 0.64;5.69 | 0.96;4.32 |
|  | Percentiles 25^th^, 75^th^ | 0.67;14.49 | 0.71;8.41 | 0.24;7.58 | 0.51;6.22 |
|  | Min-Max | 0.04;81.62 | 0-145.71 | 0-99.37 | 0.02-41.24 |
| *w*OvFtsZ/ OvActin | %Change^b^ | -146.59 | -29.55 | 17.61 | 0.00 |

**S20 Table: Effect of treatment on the presence of Wolbachia in nodule sections: PCR^a,^—ITT-analysis**

*N=Number; CI=Confidence interval; TA=Treatment arm; ITT=Intention-to-treat; RIF=Rifampicin; ALB=Albendazole*

*^a^For this analysis, qPCR results from nodules with dead worms only or without an actin signal were excluded*

*^b^Percentage change was calculated by using the No Treatment/Control arm as reference*

*Descriptive statistics (median, minimum, maximum, percentiles, 95% CI, and percentage change) were calculated using Stata version 16*
