# Supplementary material for: Assessing the efficacy of high-dose rifampicin plus albendazole against onchocerciasis
Source: PLoS Negl Trop Dis. 2026 Aug 12;20(8):e0014594. doi: 10.1371/journal.pntd.0014594 (PMC13492750; doi:10.1371/journal.pntd.0014594)
Supplement: Supplementary file 21 [file pntd.0014594.s027.docx]

**S21** T**able: Effect of treatment on the presence of Wolbachia in nodule sections: statistics^a,b^—ITT-analysis**

| **Comparison** | **wOvFtsZ** | **OvActin** | **wOvFtsZ/OvActin** |
| --- | --- | --- | --- |
| TA1 vs TA2 | OR 1.5200 [0.84;2.74]  *p* = 0.1631 | OR 1.2647 [0.69;2.31]  *p* = 0.4442 | OR 1.1500 [0.80;1.66]  *p* = 0.4562 |
| TA1 vs TA3 | OR 1.6134 [1.00; 2.61]  *p* = 0.0522 | OR 1.0025 [0.62;1.63]  *p* = 0.9920 | OR 1.2803 [0.88;1.87]  *p* = 0.1984 |
| TA1 vs TA 4 | OR 1.3796 [0.94;2.02]  *p* = 0.0986 | OR 1.0181 [0.69; 1.49]  *p* = 0.9266 | OR 1.2333 [0.88;1.71]  *p* = 0.2079 |
| TA2 vs TA3 | OR 1.0632 [0.54;2.08]  *p* = 0.8580 | OR 0.7933 [0.40;1.56]  *p* = 0.5025 | OR 1.1134 [0.75;1.66]  *p* = 0.5957 |
| TA2 vs TA4 | OR 0.9101 [0.50;1.66]  *p* = 0.7579 | OR 0.8078 [0.44;1.49]  *p* = 0.4921 | OR 1.0718 [0.76;1.52]  *p* = 0.6969 |
| TA3 vs TA4 | OR 0.8561 [0.52;1.42]  *p* = 0.5484 | OR 1.0122 [0.62; 1.66]  *p =* 0.9616 | OR 0.9639 [0.67;1.38]  *p* = 0.8407 |

*TA=Treatment arm; ITT=Intention-to-treat; TA1=RIF+ALB- 14 days; TA2=RIF+ALB- 7 days; TA3=ALB alone- 14 days; TA4=No treatment/control*

*^a^Alternating linear regression after log10-transformation (0.1 was added to all values to circumvent zero values)*

*^b^Odds ratios (OR) showing the presence of Wolbachia: pairwise comparisons between treatment groups as specified in the ‘Comparison’ column.*

*Alternating linear regression calculated using SAS^®^ version 9.4*
