# Supplementary material for: Assessing the efficacy of high-dose rifampicin plus albendazole against onchocerciasis
Source: PLoS Negl Trop Dis. 2026 Aug 12;20(8):e0014594. doi: 10.1371/journal.pntd.0014594 (PMC13492750; doi:10.1371/journal.pntd.0014594)
Supplement: Supplementary file 22 [file pntd.0014594.s028.docx]

**S22 Table: Changes in microfilarial densities 4-, 18-, and 20-** **months following treatment —ITT analysis**

|  | | **RIF+ALB –**  **14 Days (TA1)** | **RIF+ALB –**  **7 Days (TA2)** | **ALB alone – 14 Days (TA3)** | **No Treatment/**  **Control (TA4)** | **P-value** |
| --- | --- | --- | --- | --- | --- | --- |
| **Baseline** |  | | | | | |
| Participants | N | 30 | 30 | 30 | 30 |  |
| MF Positive | N (%) | 30 (100) | 30 (100) | 30 (100) | 30 (100) |  |
| MF/mg skin snip | Median | 0.70 | 0.83 | 1.06 | 0.77 | 0.581^a^ |
|  | Min-Max | 0.06-15.75 | 0.08-15.81 | 0.10-64.73 | 0.06-34.71 |  |
|  | Percentiles 25^th^- 75^th^ | 0.33;1.17 | 0.33;2.87 | 0.36;3.58 | 0.25;1.94 |  |
|  | GM | 0.63 | 0.91 | 1.19 | 0.76 |  |
|  | 95% CI of the GM | 0.36-1.09 | 0.55-1.51 | 0.66-2.13 | 0.44-1.30 |  |
| **4 Months** |  | | | | | |
| Participants | N | 30 | 28 | 29 | 28 |  |
| MF Positive | N (%) | 23 (76.7) | 23 (82.1) | 25 (86.2) | 27 (96.4) | 0.173^b^ |
| MF/mg skin snip | Median | 0.73 | 0.51 | 0.97 | 1.53 | 0.247^a^ |
|  | Min-Max | 0.00-66.28 | 0.00-6.26 | 0.00-52.46 | 0.00-33.77 |  |
|  | Percentiles 25^th^- 75^th^ | 0.16;1.83 | 0.10;1.65 | 0.18;1.76 | 0.23;3.56 |  |
|  | GM | 0.13 | 0.14 | 0.29 | 0.90 |  |
|  | 95% CI of the GM | 0.03-0.63 | 0.03-0.60 | 0.07-1.12 | 0.33-2.42 |  |
|  | P-value* | 0.784 | 0.173 | 0.039 | 0.074 |  |
| **18 Months** |  | | | | | |
| Participants | N | 27 | 28 | 30 | 28 |  |
| MF Positive | N (%) | 19 (70.4) | 22 (78.6) | 19 (63.3) | 21 (75.0) | 0.620^b^ |
| MF/mg skin snip | Median | 0.76 | 0.44 | 0.12 | 0.53 | 0.444^a^ |
|  | Min-Max | 0.00-17.20 | 0.00-16.13 | 0.00-19.17 | 0.00-11.92 |  |
|  | Percentiles 25^th^- 75^th^ | 0.00;2.08 | 0.11;1.18 | 0.00;1.28 | 0.02;2.41 |  |
|  | GM | 0.08 | 0.12 | 0.03 | 0.10 |  |
|  | 95% CI of the GM | 0.01-0.45 | 0.03-0.54 | 0.00-0.14 | 0.02-0.55 |  |
|  | P-value* | >0.999 | 0.200 | 0.013 | 0.972 |  |
| **20 Months** |  | | | | | |
| Participants | N | 26 | 27 | 30 | 27 |  |
| MF Positive | N (%) | 19 (73.1) | 22 (81.5) | 20 (66.7) | 22 (81.5) | 0.507^b^ |
| MF/mg skin snip | Median | 0.78 | 1.25 | 0.23 | 1.60 | 0.123^a^ |
|  | Min-Max | 0.00-34.90 | 0.00-12.18 | 0.00-20.56 | 0.00-26.98 |  |
|  | Percentiles 25^th^- 75^th^ | 0.00;3.02 | 0.15;4.09 | 0.00;1.27 | 0.06;6.11 |  |
|  | GM | 0.11 | 0.27 | 0.04 | 0.28 |  |
|  | 95% CI of the GM | 0.02-0.66 | 0.05-1.32 | 0.01-0.20 | 0.05-1.49 |  |
|  | P-value* | >0.999 | 0.181 | 0.014 | 0.181 |  |

*RIF=Rifampicin; ALB=Albendazole; TA=Treatment arm; N=Number; GM=Geometric mean; ITT=Intention-to-treat*

*^a^Kruskal-Wallis test*

*^b^Fisher exact test*

**Comparison to baseline (Wilcoxon signed-rank test)*

*Geometric mean adjusted by adding 0.0001 to the MF/mg skin snip values to account for zero values*

*Statistics calculated using Stata version 16*
