# Supplementary material for: Assessing the efficacy of high-dose rifampicin plus albendazole against onchocerciasis
Source: PLoS Negl Trop Dis. 2026 Aug 12;20(8):e0014594. doi: 10.1371/journal.pntd.0014594 (PMC13492750; doi:10.1371/journal.pntd.0014594)
Supplement: Supplementary file 23 [file pntd.0014594.s029.docx]

**S23 Table: Iron deposition in adult worm—PP analysis**

| **Treatment group** | **Number of Patients/**  **Nodules^a^** | **Number of living female worms**  ***p = 0.373^b^*** | | | | **Number of living male worms**  ***p = 0.872*** | | | |
| --- | --- | --- | --- | --- | --- | --- | --- | --- | --- |
|  |  | **Litle iron (+)** | **Much iron (++)** | **Not judgeable** | **Total** | **Litle iron (+)** | **Much iron (++)** | **Not judgeable** | **Total** |
| RIF+ALB –  14 Days (TA1) | 17/38 | 1 (1.5) | 29 (42.0) | 39 (56.52) | 69 | 1 (3.6) | 2 (7.1) | 25 (89.3) | 28 |
| RIF+ALB –  7 Days (TA2) | 21/47 | 0 (0.0) | 45 (54.2) | 38 (45.8) | 83 | 1 (2.9) | 5 (14.3) | 29 (82.9) | 35 |
| ALB alone – 14 Days (TA3) | 20/40 | 1 (1.6) | 27 (44.3) | 33 (54.1) | 61 | 0 (0.0) | 4 (14.8) | 23 (85.2) | 27 |
| No Treatment/  Control (TA4) | 21/43 | 2 (3.0) | 36 (53.7) | 29 (43.3) | 67 | 2 (6.7) | 4 (13.3) | 24 (80.0) | 30 |
| Total | 79/168 | 4 (1.4) | 137 (48.9) | 139 (49.6) | 280 | 4 (3.3) | 15 (12.5) | 101 (84.2) | 120 |

*TA=Treatment arm; PP=per-protocol; RIF=Rifampicin; ALB=Albendazole; CI=Confidence interval*

*^a^Only evaluable patients/nodules are included: Out of the 191 nodules from 89 patients (PP), 23 nodules from 10 patients were not evaluable (non-onchocercal origin, e.g., foreign body granulomas, lipomas or lymph nodes: TA1 = 8, TA2 = 5, TA3 = 9, TA4 = 1)*

*^b^Fisher`s exact test*
