# Supplementary material for: Assessing the efficacy of high-dose rifampicin plus albendazole against onchocerciasis
Source: PLoS Negl Trop Dis. 2026 Aug 12;20(8):e0014594. doi: 10.1371/journal.pntd.0014594 (PMC13492750; doi:10.1371/journal.pntd.0014594)
Supplement: Supplementary file 24 [file pntd.0014594.s030.docx]

**S24 Table: Iron deposition in adult worm—ITT analysis**

| **Treatment group** | **Number of Patients/**  **Nodules^a^** | **Number of living female worms**  ***p = 0.227^b^*** | | | | **Number of living male worms**  ***p = 0.496*** | | | |
| --- | --- | --- | --- | --- | --- | --- | --- | --- | --- |
|  |  | **Litle iron (+)** | **Much iron**  **(++)** | **Not judgeable** | **Total** | **Litle iron (+)** | **Much iron (++)** | **Not judgeable** | **Total** |
| RIF+ALB –  14 Days (TA1) | 20/45 | 1 (1.3) | 31 (40.3) | 45 (58.4) | 77 | 1 (2.9) | 2 (5.9) | 31 (91.2) | 34 |
| RIF+ALB –  7 Days (TA2) | 22/52 | 0 (0.0) | 49 (55.1) | 40 (44.9) | 89 | 2 (5.3) | 6 (15.8) | 30 (79.0) | 38 |
| ALB alone – 14 Days (TA3) | 25/48 | 1 (1.4) | 33 (46.5) | 37 (52.1) | 71 | 0 (0.0) | 6 (20.0) | 24 (80.0) | 30 |
| No Treatment/  Control (TA4) | 25/51 | 2 (2.7) | 41 (54.7) | 32 (42.7) | 75 | 2 (5.9) | 6 (17.7) | 26 (76.5) | 34 |
| Total | 92/196 | 4 (1.3) | 154 (49.4) | 154 (49.4 | 312 | 5 (3.7) | 20 (14.7) | 111 (81.6) | 136 |

*TA=Treatment arm; ITT=Intention-to-treat; RIF=Rifampicin; ALB=Albendazole; CI=Confidence interval*

*^a^Only evaluable patients/nodules are included: Out of the 223 nodules from 105 patients (ITT), 27 nodules from 13 patients were not evaluable (non-onchocercal origin e.g., foreign body granulomas, lipomas or lymph nodes: TA1 = 7, TA2 = 9, TA3 = 10, TA4 = 1)*

*^b^Fisher`s exact test*
