# Supplementary material for: Assessing the efficacy of high-dose rifampicin plus albendazole against onchocerciasis
Source: PLoS Negl Trop Dis. 2026 Aug 12;20(8):e0014594. doi: 10.1371/journal.pntd.0014594 (PMC13492750; doi:10.1371/journal.pntd.0014594)
Supplement: Supplementary file 25 [file pntd.0014594.s031.docx]

**S25 Table: Viability status of adult worm —PP analysis**

| **Treatment group** | **Number of Patients/**  **Nodules^a^** | **Number of female worms** | | | | | | **Number of male worms^b^** | | | |
| --- | --- | --- | --- | --- | --- | --- | --- | --- | --- | --- | --- |
|  |  | **Viable (non-degenerated)** | **Neoplasm** | **Calcified** | **Resorbed** | **Others*** | **Total** | **Viable (non-degenerated)** | **Calcified** | **Others*** | **Total** |
| RIF+ALB –  14 Days (TA1) | 17/38 | 39 (42.4) | 4 (4.4) | 36 (39.1) | 5 (5.4) | 8 (8.7) | 92 | 16 (51.6) | 13 (41.9) | 2 (6.5) | 31 |
| RIF+ALB –  7 Days (TA2) | 21/47 | 42 (35.3) | 3 (2.5) | 48 (40.3) | 10 (8.4) | 16 (13.5) | 119 | 22 (61.1) | 14 (38.9) | 0 (0.0) | 36 |
| ALB alone – 14 Days (TA3) | 20/40 | 42 (46.2) | 3 (3.3) | 32 (35.2) | 6 (6.6) | 8 (8.8) | 91 | 15 (51.7) | 14 (48.3) | 0 (0.0) | 29 |
| No Treatment/  Control (TA4) | 21/43 | 34 (32.4) | 5 (4.8) | 46 (43.8) | 11 (10.5) | 9 (8.6) | 105 | 15 (51.7) | 14 (48.3) | 0 (0.0) | 29 |
| Total | 79/168 | 157 (38.6) | 15 (3.7) | 162 (39.8) | 32 (7.9) | 41 (10.1) | 407 | 75 (59.1) | 49 (38.6) | 3 (2.4) | 127 |

*^a^Only evaluable patients/nodules are included: Out of the 191 nodules from 89 patients (PP), 23 nodules from 10 patients were not evaluable (non-onchocercal origin, e.g., foreign body granulomas, lipomas or lymph nodes: TA1 = 8, TA2 = 5, TA3 = 9, TA4 = 1)*

**Other categories include: neoplasm plus calcified, neoplasm plus resorbed, neoplasm plus calcified plus resorbed and calcified plus resorbed*

*^b^No male worm exhibited neoplasm or resorbed alone*
