# Supplementary material for: Assessing the efficacy of high-dose rifampicin plus albendazole against onchocerciasis
Source: PLoS Negl Trop Dis. 2026 Aug 12;20(8):e0014594. doi: 10.1371/journal.pntd.0014594 (PMC13492750; doi:10.1371/journal.pntd.0014594)
Supplement: Supplementary file 26 [file pntd.0014594.s032.docx]

| **Treatment group** | **Number of Patients/**  **Nodules^a^** | **Number of female worms** | | | | | | **Number of male worms^b^** | | | |
| --- | --- | --- | --- | --- | --- | --- | --- | --- | --- | --- | --- |
|  |  | **Viable (non-degenerated)** | **Neoplasm** | **Calcified** | **Resorbed** | **Others*** | **Total** | **Viable (non-degenerated)** | **Calcified** | **Others** | **Total** |
| RIF+ALB –  14 Days (TA1) | 20/45 | 45 (43.3) | 4 (3.9) | 37 (35.6) | 8 (7.7) | 10 (9.6) | 104 | 20 (54.1) | 15 (40.5) | 2 (5.4) | 37 |
| RIF+ALB –  7 Days (TA2) | 22/52 | 48 (38.1) | 3 (2.4) | 48 (38.1) | 11 (8.7) | 16 (12.7) | 126 | 24 (60.0) | 16 (40.0) | 0 (0.0) | 40 |
| ALB alone – 14 Days (TA3) | 25/48 | 49 (45.8) | 4 (3.7) | 36 (33.6) | 7 (6.5) | 11 (10.3) | 107 | 17 (51.5) | 16 (48.5) | 0 (0.0) | 33 |
| No Treatment/  Control (TA4) | 25/51 | 41 (36.0) | 5 (4.4) | 46 (40.4) | 12 (10.5) | 10 (8.8) | 114 | 25 (71.4) | 8 (22.9) | 2 (5.7) | 35 |
| Total | 92/196 | 183 (40.6) | 16 (3. 6) | 167 (37.0) | 38 (8.4) | 47 (10.4) | 451 | 86 (59.3) | 55 (37.9) | 4 (2.8) | 145 |

**S26 Table: Viability status of adult worm—ITT analysis**

*TA=Treatment arm; ITT=Intention-to-treat; RIF=Rifampicin; ALB=Albendazole; CI=Confidence interval*

*^a^Only evaluable patients/nodules are included: Out of the 223 nodules from 105 patients (ITT), 27 nodules from 13 patients were not evaluable (non-onchocercal origin e.g., foreign body granulomas, lipomas or lymph nodes: TA1 = 7, TA2 = 9, TA3 = 10, TA4 = 1)*

**Other categories include: neoplasm plus calcified, neoplasm plus resorbed, neoplasm plus calcified plus resorbed and calcified plus resorbed*

*^b^No male worm exhibited neoplasm or resorbed alone*
